# Irlactane and Tremulane Sesquiterpenes from the Cultures of the Medicinal Fungus *Irpex lacteus* HFG1102

**DOI:** 10.1007/s13659-020-00239-z

**Published:** 2020-04-10

**Authors:** He-Ping Chen, Xu Ji, Zheng-Hui Li, Tao Feng, Ji-Kai Liu

**Affiliations:** 1 School of Pharmaceutical Sciences, South-Central University for Nationalities, Wuhan, 430074 People’s Republic of China; 2 School of Chemical Science and Technology, Yunnan University, Kunming, 650091 People’s Republic of China

**Keywords:** *Irpex lacteus*, Meruliaceae, Irlactane, Tremulane, Sesquiterpene

## Abstract

**Electronic supplementary material:**

The online version of this article (10.1007/s13659-020-00239-z) contains supplementary material, which is available to authorized users.

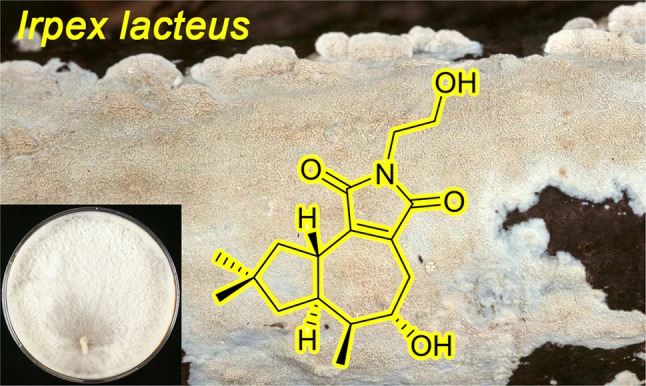

## Introduction

Medicinal fungi are dominate resources of natural products with diverse structural scaffolds and promising biological activities [1]. The wood-decaying fungus *Irpex lacteus* widely distributed throughout temperate areas globally is traditionally used as a medicinal fungus for diuretic, anti-bacterial, and anti-inflammatory function. The *Yishenkang* capsule made from the fermentated polysaccharide fraction of this fungus is clinically used as a remedy for chronic glomerulonephritis in China [2]. However, the fungus has not yet been fully chemically explored.

**Fig. 1:**
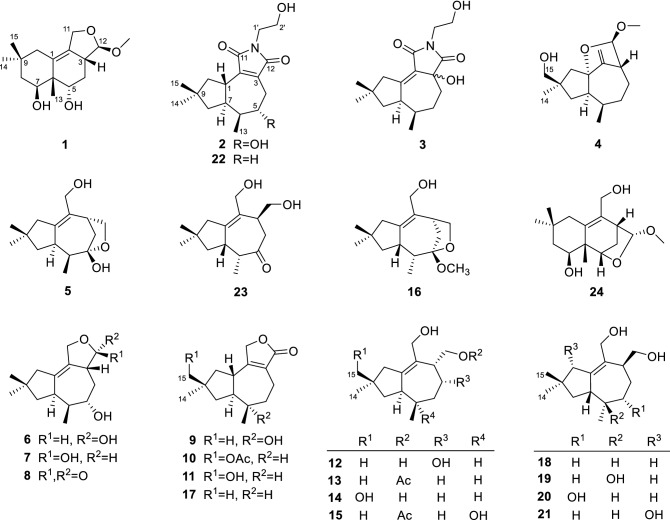
Structures of compounds **1**–**24**

Tremulanes are a class of 5/7-ring-fused sesquiterpenoid which are typically found from fungi [1], and were originally isolated from the wood-decaying fungus *Phellinus tremulae* [3], and later from the medicinal fungus *Phellinus igniarius*, and the mushroom *Conocybe siliginea*. Recently, we reported ten tremulane/tremulane-related sesquiterpenoids, namely irlactins A–J from the cultures of the fungus *I. lacteus*, among which irlactins A–D featured by an unprecedented skeleton in sesquiterpenoid class [4–6]. Herein, as part of our ongoing research for secondary metabolites as promising drug leads from higher fungi, we reported fifteen previously undescribed irlactane/tremulanes, namely irlactin K (**1**), and irpexolactins A–N (**2**–**15**) (Fig. 1), along with nine known ones from the culture broth of the fungus *I. lacteus*.

## Results and Discussion

### Structure Elucidation of Previously Undescribed Fungal Metabolites (1–15)

**Fig. 2:**
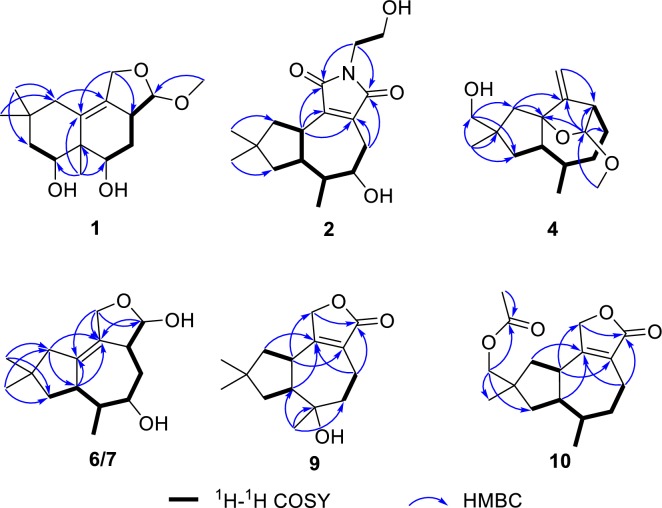
Key ^1^H-^1^H COSY and HMBC correlations of compounds **1**, **2**, **4**, **6**/**7**, **9**, and **10**

**Fig. 3:**
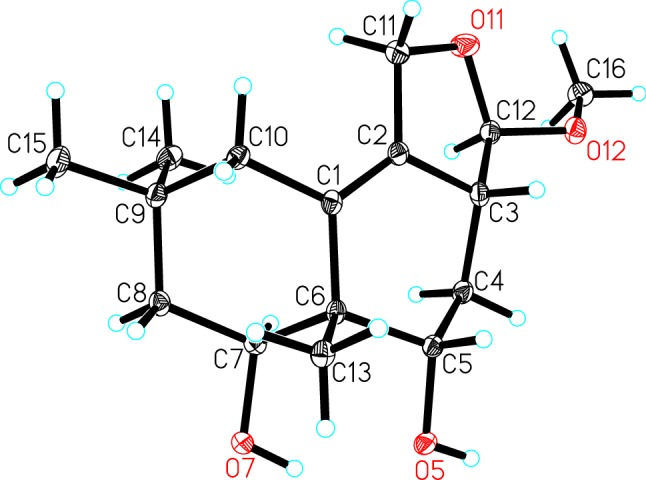
ORTEP drawing of compound **1**

Irlactin K (**1**) was isolated as colorless crystals. The ^1^H NMR spectroscopic data (Table 1) presented three methyl singlets (*δ*_H_ 0.88, 1.00, 1.23) and a methoxy group (*δ*_H_ 3.44). The ^13^C NMR spectrum (Table 4) showed sixteen carbon resonances ascribable to four methyls, four methylenes (one oxygenated), four methines (three oxygenated), and four quaternary carbons. All these data are reminiscent of those of irlactins C and D which were isolated from the cultures of the same fungus but different strains [4]. The presence of a methoxy group rather than a hydroxy group at C-12 in **1** as suggested by the HMBC correlation from the methoxy at *δ*_H_ 3.44 to the ketal carbon at *δ*_C_ 111.3 (C-12) discriminated its structure from those of irlactins C/D. The above assignments led to the determination of the planar structure of **1** as depicted in Fig. 2. The absolute configuration of **1** was determined to be 3*R*,5*S*,6*R*,7*S*,12*R* via X-ray single crystal diffraction analysis with the small Flack parameter 0.15(6) (Fig. 3).

**Table 1:** ^1^H NMR spectroscopic data of compounds **1**–**5** (600 MHz, *δ* in ppm)

No	**1**^a^	**2**^a^	**3**^b^	**4**^c^	**5**^a^
1		2.83, overlapped			
3	2.45, m			2.66, dd (3.7, 3.7)	2.98, dd (7.4, 7.4)
4	1.99, ddd (11.6, 5.0, 3.6) 1.59, dd (11.6, 11.6)	2.81, overlapped 2.65, ddd (17.3, 3.0, 3.0)	1.98, ddd (14.5, 4.2, 2.5) 1.76, dd (14.5, 3.0)	1.77, dddd (13.7, 13.7, 3.7, 3.7) 1.68, overlapped	2.00, d (12.3) 1.83, dd (12.3, 7.4)
5	3.76, dd (11.6, 3.6)	3.63, overlapped	2.44, dddd (14.4, 14.4, 3.3, 3.3) 1.74, m	1.95, m 1.59, m	
6		1.98, m	1.96, m	2.10, m	1.92, m
7	4.07, dd (11.2, 5.3)	2.59, m	3.59, m	2.40, ddd (14.3, 6.6, 6.6)	3.25, m
8	1.53, dd (13.5, 11.2) 1.48, dd (13.5, 5.3)	1.49, dd (14.2, 1.5) 1.45, d (14.2)	1.65, br dd (12.5, 8.4) 1.45, dd (12.5, 11.0)	1.67, overlapped 1.14, dd (12.5, 6.0)	1.51, dd (12.1, 12.1) 1.46, dd (12.1, 8.5)
10	1.96, br d (13.5) 1.78, br d (13.5)	2.32, dd (12.9, 7.6) 1.54, dd (12.9, 10.9)	3.25, d (17.8) 2.19, d (17.8)	2.17, d (14.8) 1.65, d (14.8)	2.30, br d (15.7) 1.99, br d (15.7)
11	4.31, br s			5.13, s 4.94, s	4.07, d (12.1) 3.95, d (12.1)
12	4.64, d (6.0)			4.62, s	4.32, dd (7.8, 7.4) 3.84, d (7.8)
13	1.23, s	1.03, d (7.0)	0.84, d (7.0)	0.89, d (7.0)	0.87, d (7.0)
14	0.88, s	1.07, s	0.89, s	1.10, s	1.11, s
15	1.00, s	1.07, s	1.17, s	3.39, d (10.0) 3.37, d (10.0)	0.94, s
1′		3.56, t (5.6)	3.78, t (5.2)		
2′		3.63, t (5.6)	3.83, t (5.2)		
2′-OH			2.57, br s		
-OMe	3.44, s			3.19, s	

^a^Recorded in CD_3_OD

^b^Recorded in CDCl_3_

^c^Recorded in acetone-*d*_6_

Irpexolactin A (**2**) was obtained as a pale-yellow oil. The molecular formula C_17_H_25_O_4_N with six degrees of unsaturation was deduced from the sodium adduct ion peak at *m/z* 330.1685 [M + Na]^+^ in the HRESIMS analysis (calcd for C_17_H_25_O_4_NNa, 330.1676). The ^1^H and ^13^C NMR data of **2** (Tables 1, 4) revealed the presence of three methyls (two singlets at *δ*_H_ 1.07, 1.07, one doublet at *δ*_H_ 1.03), five methylenes (one oxygenated at *δ*_C_ 60.4), four methines (one oxygenated at *δ*_C_ 74.7), and five quaternary carbons (two carbonyls at *δ*_C_ 172.7, C-11; 173.2, C-12) and a tetrasubstituted double bond at *δ*_C_ 145.5 (C-2), 139.4 (C-3), indicating three double bond equivalences. All these data bore striking similarity with those of coriolopsin A, a tremulane sesquiterpene isolated from the endophytic fungus *Coriolopsis* sp. J5 [7]. The structure of compound **2** was further corroborated by 2D NMR analysis, which suggested that the Δ^1^ double bond in coriolopsin A shifted to Δ^2^ in **2** by HMBC correlation from H-1 (*δ*_H_ 2.83), H_2_-4 (*δ*_H_ 2.81, 2.65) to C-2 and C-3 (Fig. 2). Furthermore, the oxygenated methine at *δ*_C_ 74.7 was assigned to C-5 by the HMBC correlation from H_3_-13 (*δ*_H_ 1.03) to C-5 and ^1^H-^1^H COSY correlation between H-5 (*δ*_H_ 3.63) and H-6 (*δ*_H_ 1.98) (Fig. 2). The remaining two methylenes was assigned to an aminoethanol group same as that harbored in vibralactamide A [8] according to the chemical shifts of C-1′ (*δ*_C_ 41.3), C-2′ (*δ*_C_ 60.4) and the ^1^H-^1^H COSY correlations between H-1′ (*δ*_H_ 3.56)/H-2′ (*δ*_H_ 3.63). The HMBC correlations from H-1′ to two carbonyls at C-11 and C-12, revealed a succinamide moiety.

**Fig. 4:**
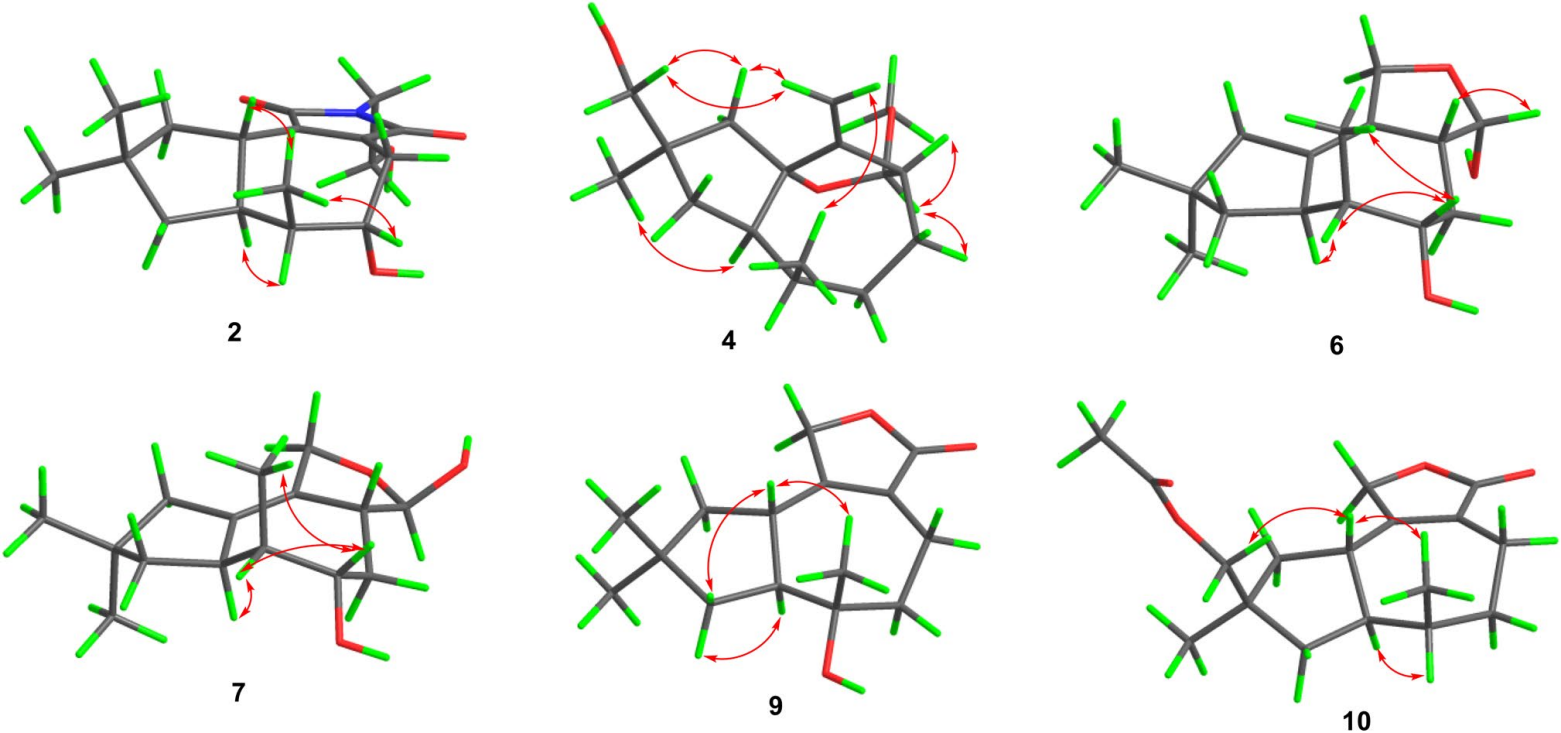
Key ROESY correlations of compounds **2**, **4**, **6**/**7**, **9**, and **10**

Compound **2** possessed a 1-(2-hydroxyethyl)pyrrolidine-2,5-dione unit which is unusual in sesquiterpenoid family. The relative configuration of **2** was ascertained by the ROESY spectrum. The ROESY correlations of H-1/H_3_-13/H-5, and H-6/H-7 demonstrated that when 5-OH and H-7 occupied *α* orientation, H-1 and Me-13 would be *β* orientation (Fig. 4). All the above assignments successfully constructed the structure of **2** (Fig. 1) and irpexolactin A was given as the trivial name.

The colorless oil **3** had a molecular formula of C_17_H_25_NO_4_ identical with that of **2** as suggested by HRESIMS result. The ^13^C NMR data of irlactam (**22)** [6] and **3** (Table 4) displayed remarkable disparities of C-1, C-2, and C-3, suggesting their main differences located at C-1 to C-3. The HMBC correlations from H-7 (*δ*_H_ 3.59), H-10 (*δ*_H_ 3.25 and 2.19) to C-1 (*δ*_C_ 170.1), C-2 (*δ*_C_ 123.3), and from H-4 (*δ*_H_ 1.98 and 1.76) to C-2, C-3 (*δ*_C_ 74.4) enabled the assignments of double bond between C-1 and C-2, and a hydroxy attached to C-3. The ROESY correlations between H-6 (*δ*_H_ 1.96) and H-7 revealed that the *β* orientation of Me-13 and *α* orientation of H-7. However, the configuration of 3-OH remained undetermined due to the shortage of sample for making any chemical derivatives. Thus, the structure of compound **3** was determined as shown in Fig. 1, and was named irpexolactin B.

Compound **4** was obtained as a colorless oil. The HRESIMS experiment gave a sodium adduct ion peak at *m/z* 289.1774 [M + Na]^+^, corresponding to a molecular formula of C_16_H_26_O_3_ (calcd for C_16_H_26_O_3_Na, 289.1774) with four double bond equivalences. The 1D NMR data (Tables 1, 4) displayed three methyls (one methoxy), six methylenes (one oxygenated, a terminal double bond), four methines (one acetal methine), and three quaternary carbons (one oxygenated, one embedded into a terminal double bond). All these data are reminiscent of the known compound 1*β*,12-epoxy-14-hydroxy-2(11)-tremulene [9]. The HMBC correlations from H-12 (*δ*_H_ 4.62) to C-1 (*δ*_C_ 93.7), C-2 (*δ*_C_ 156.1), C-3 (*δ*_C_ 52.2), and C-4 (*δ*_C_ 26.7), and from the methoxy at *δ*_H_ 3.19 to C-12 (*δ*_C_ 106.2) indicated that the methylene C-12 in 1*β*,12-epoxy-14-hydroxy-2(11)-tremulene was substituted by a methoxy to give **4** (Fig. 2). The ROESY correlations of H_3_-13 (*δ*_H_ 0.89)/H_2_-11 (*δ*_H_ 5.13, 4.94), H_2_-15 (*δ*_H_ 3.39, 3.37)/H-11a, and H_3_-14 (*δ*_H_ 1.10)/H-7 (*δ*_H_ 2.40) demonstrated the *β* orientations for Me-13 and 15-CH_2_OH (Fig. 4), which is different to that of 1*β*,12-epoxy-14-hydroxy-2(11)-tremulene. The key ROESY correlations of H-12 (*δ*_H_ 4.62)/H-3 (*δ*_H_ 2.66), H-12/H-4*β* (*δ*_H_ 1.68) (Fig. 4) helped to assign the relative configuration of C-12 as R* and *α* oriented. Thus, compound **4** was established as irpexolactin C.

Compounds **5** and **23** were inseparable mixture with a ratio of 1:1.6. HRESIMS results revealed that they had the same molecular formula as C_15_H_24_O_3_ (*m/z* 275.1624 [M + Na]^+^, calcd for C_15_H_24_O_3_Na, 275.1618). The NMR spectroscopic analysis of the mixture led to the identification of 11,12-dihydroxy-1-tremulen-5-one (**23**), a known tremulane sesquiterpenoid isolated from the mushroom *Conocybe siliginea* [10]. The remaining spectroscopic signals were ascribable to three methyls, five methylenes (two oxygenated), three methines, and four quaternary carbons (two olefinic, one ketal carbon) (Tables 1, 4). All these data showed resemblance with those of conocenol C (**16**) [11], except substituted patterns at C-5. In compound **5**, a hydroxy group was attached to C-5, while in conocenol C a methoxy instead, which was deferred by HMBC correlation from H-12 (*δ*_H_ 4.32 and 3.84) to C-5 (*δ*_C_ 112.8) and the HRESIMS data. Notably, the inseparable mixture of **5** and **23** seems to be the interconversion of hemiketal form **5** and ketone form **23**. Therefore, compound **5** was identified as irpexolactin D (shown in Fig. 1).

Compounds **6** and **7** were inseparable mixture with a ratio of 1.9:1 according to the integration of ^1^H NMR spectrum. The HRESIMS analysis demonstrated that they had the same molecular formula of C_15_H_24_O_3_ (*m/z* 275.1621 [M + Na]^+^, calcd for 275.1618). The doubled signals in the ^1^H and ^13^C NMR spectra and similar proton peak types indicated that the two compounds were epimers. The 1D NMR spectra of the mixture showed signals which were categorized into three methyls, four methylenes (one oxygenated), five methines (two oxygenated), and three quaternary carbons (two olefinic) (Tables 2, 4). The data showed a resemblance to those of ceriponol G [12], except an absence of a methoxy signal at C-12. Thus, the planar structures of **6** and **7** were elucidated as shown in Fig. 2. The inseparable feature of **6** and **7** implied that they were semiacetal tautomers. The 12-OH of **6** and **7** were assigned as *α* and *β* orientation, respectively, by the key ROESY cross peaks between H-3 (*δ*_H_ 2.54)/H-12 (*δ*_H_ 5.23) of **6**, and the absence of ROESY signals between H-3 (*δ*_H_ 2.27) and H-12 (*δ*_H_ 4.74) of **7** (Fig. 4). The 5-OH of **6** and **7** were *α* orientation as suggested by the ROESY correlations between H_3_-13/H-5 (Fig. 4). Therefore, compounds **6** and **7** were identified as irpexolactins E and F.

**Table 2:** ^1^H NMR spectroscopic data of compounds **6**–**11** (600 MHz, *δ* in ppm)

No	**6**^a^	**7**^a^	**8**^a^	**9**^a^	**10**^b^	**11**^b^
1				2.84, ddd (13.3, 13.3, 10.5)	2.78, ddd (13.3, 13.3, 10.5)	2.76, ddd (13.3, 13.3, 10.5)
3	2.54, m	2.27, m	3.64, br d (12.9)			
4	1.98, br dd (13.5, 6.3) 1.83, ddd (13.5, 13.5, 10.6)	2.08, m 1.71, ddd (13.5, 13.5, 10.6)	2.00, ddd (14.2, 5.0, 2.8) 1.84, ddd (14.2, 12.9, 2.0)	2.53, ddd (17.2, 8.1, 2.5) 2.20, ddd (17.2, 10.6, 2.6)	2.52, br d (17.0) 2.32, br dd (17.0, 2.6)	2.51, ddd (17.0, 4.0, 4.0) 2.33, ddd (17.0, 12.0, 4.0)
5	3.75, dd (10.6, 5.0)	3.74, dd (10.6, 5.0)	3.97, ddd (5.0, 5.0, 2.0)	1.88, ddd (14.0, 8.1, 2.6) 1.69, ddd (14.0, 10.6, 2.5)	1.75, m 1.68, m	1.74, m 1.68, m
6	1.93, m	1.93, m	1.87, m		2.15, overlapped	2.16, overlapped
7	3.38, m	3.37, m	3.45, m	2.25, ddd (10.5, 10.5, 8.6)	2.13, overlapped	2.13, overlapped
8	1.47, dd (12.3, 1.6) 1.44, d (12.3)	1.47, dd (12.3, 1.6) 1.44, d (12.3)	1.52, dd (13.6, 12.1) 1.48, d (13.6)	1.73, dd (13.0, 8.6) 1.58, dd (13.0, 10.5)	1.56, overlapped 1.45, dd (13.0, 7.5)	1.55, dd (12.7, 12.7) 1.39, dd (12.7, 7.1)
9						
10	2.04, overlapped 1.92, overlapped	2.05, overlapped 1.89, overlapped	1.93, br s	1.82, dd (12.2, 7.6) 1.50, dd (12.2,12.2)	1.97, dd (12.7, 7.7) 1.33, dd (12.2, 12.2)	2.02, dd (12.2, 7.6) 1.27, dd (12.2, 12.2)
11	4.39, ddd (13.0, 5.0, 2.5) 4.18, ddd (13.0, 3.6, 2.2)	4.32, ddd (13.0, 3.5, 2.0) 4.20, ddd (13.0, 4.5, 2.5)	4.80, br d (13.5) 4.72, br d (13.5)	4.77, d (18.0) 4.70, d (18.0)	4.63, br d (17.0) 4.56, br d (17.0)	4.64, d (16.9) 4.56, d (16.9)
12	5.23, d (5.0)	4.74, d (7.4)				
13	0.91, d (7.6)	0.92, d (7.6)	0.88, d (7.0)	1.24, s	0.95, d (7.6)	0.95, d (7.0)
14	1.09, s	1.09, s	0.91, s	1.09, s	1.11, s	1.09, s
15	1.05, s	1.03, s	1.13, s	1.08, s	3.87, d (10.5) 3.81, d (10.5)	3.40, d (10.7) 3.37, d (10.7)
AcO-					2.09, s	

^a^Recorded in CD_3_OD

^b^Recorded in CDCl_3_

The colorless oil of compound **8** possessed a molecular formula of C_15_H_22_O_3_, suggested by HRESIMS analysis (*m/z* 273.1468 [M + Na]^+^, calcd for C_15_H_22_O_3_Na, 273.1461). The ^1^H and ^13^C NMR data of **8** (Tables 2, 4) are similar to those of **6**/**7**, suggesting that **8** is congener of **6**/**7**. Elucidating the 2D NMR spectra of **8** suggested that C-12 in **8** was substituted by a carbonyl as supported by the HMBC correlations from H-4 (*δ*_H_ 2.00 and 1.84) and H-11 (*δ*_H_ 4.80 and 4.72) to C-12 (*δ*_C_ 182.6). The relative configuration of **8** was consistent with that of **6**/**7** by the ROESY analysis. Thus, the chemical structure of compound **8** was established as shown in Fig. 1 and the name irpexolactin G.

Irpexolactin H (**9**) was isolated as a colorless oil. The 1D NMR spectra (Tables 2, 5) displayed three methyl singlets, five methylenes (one oxygenated), two methines, and five quaternary carbons (two sp^3^ ones, and three sp^2^ ones). The 1D NMR data of **9** showed similarity with those of ceriponol C [12], indicating they were structurally related. Interpreting of the 2D NMR spectra of **9** suggested that the hydroxy group was substituted at C-6 in **9** instead of C-8 in ceriponol C, which supported by HMBC correlations from H_3_-13 (*δ*_H_ 1.24) to C-5 (*δ*_C_ 43.0), C-6 (*δ*_C_ 75.0), and C-7 (*δ*_C_ 55.7), from H-4 (*δ*_H_ 2.53 and 2.20) and H-8 (*δ*_H_ 1.73 and 1.58) to C-6. The relative configuration of H-1, 6-OH, H-7 were assigned as *β*, *α*, *α*, respectively, based on the ROESY signals of H-1 (*δ*_H_ 2.84)/H_3_-13/H-8*β* (*δ*_H_ 1.58), and H-7 (*δ*_H_ 2.25)/H-8*α* (*δ*_H_ 1.73) (Fig. 4). All these assignments led to the establishment of the structure of **9** as shown in Fig. 3, which was consistent with the HRESIMS results (*m/z* 273.1465 [M + Na]^+^, calcd for C_15_H_22_O_3_Na, 273.1461). Thus, compound **9** was identified as 6*α*-hydroxy-tremul-2-en-12,11-olide.

The oily compounds irpexolactins I (**10**) and J (**11**) were determined to possess the molecular formulas of C_17_H_24_O_4_ and C_15_H_22_O_3_, respectively. The 1D NMR data of **10** (Tables 2, 5) resembled to those of the co-isolate tremulenolide D (**17**) [13], with the main difference located at C-14. The HMBC correlations from H_3_-14 (*δ*_H_ 1.11 for **10**, 1.09 for **11**) to C-15 (*δ*_C_ 72.2 for **10**, 71.4 for **11**), from H-15 (*δ*_H_ 3.87, 3.81 for **10**) and a methyl singlet at *δ*_H_ 2.09 to a carbonyl at *δ*_C_ 171.5 suggested that H_3_-15 in tremulenolide D (**17**) was oxygenated into a hydroxymethyl to give **11**, which was further acetylated to yield **10** (Fig. 1). The oxygen-bearing C-15 was assigned as *β* orientation which evidenced by the ROESY correlation between H-15 (*δ*_H_ 3.87, 3.81 for **10**, 3.40, 3.37 for **11**) and H-1 (*δ*_H_ 2.78 for **10**, 2.76 for **11**) (Fig. 4), and the relative configurations of Me-13 and H-7 were identical with those of compound **9**. Therefore, the structures of **10** and **11** were established as shown in Fig. 1.

Irpexolactin K (**12**) was obtained as a colorless oil. It was assigned the molecular formula of C_15_H_26_O_3_ on the basis of HRESIMS (*m/z* 277.1781 [M + Na]^+^, calcd for C_15_H_26_O_3_Na, 277.1774). The NMR spectra of **12** (Tables 3, 5) exhibited similarities with those of the co-isolate tremulenediol A (**18**) [3]. The key difference was that a hydroxy group substituted at C-4 in **12** compared to **18**, which in accordance with the chemical formula and further confirmed by 2D NMR spectra of **12**. The ^1^H-^1^H COSY correlations between H-4 (*δ*_H_ 4.02) and H-3 (*δ*_H_ 2.80), H-5 (*δ*_H_ 1.98 and 1.85), and HMBC correlation from H-12 (*δ*_H_ 4.01, 3.84) to C-2 (*δ*_C_ 131.1), C-3 (*δ*_C_ 52.8), and C-4 (*δ*_C_ 67.5) corroborated the postulation. The relative configuration of 4-OH was determined as *α* based on the significant correlation between H-3/H-4. Hence, compound **12** was determined as shown in Fig. 1.

**Table 3:** ^1^H NMR spectroscopic data of compounds **12**–**15** (600 MHz, *δ* in ppm)

No	**12**^a^	**13**^b^	**14**^a^	**15**^a^
3	2.80, m	2.74, m	2.57, m	2.79, m
4	4.02, overlapped	1.80, ddd (14.2, 2.6, 2.6) 1.64, overlapped	1.81, overlapped 1.75, overlapped	1.85, m 1.72, overlapped
5	1.98, ddd (12.7, 12.7, 3.5) 1.85, overlapped	1.89, ddd (13.8, 3.0, 3.0) 1.61, overlapped	1.78, overlapped 1.62, m	1.95, overlapped 1.74, overlapped
6	1.88, overlapped	1.77, m	1.96, m	
7	3.07, br dd (11.3, 8.9)	3.04, br dd (9.8, 9.8)	3.10, br dd (11.7, 8.5)	3.09, dd (9.6, 9.6)
8	1.55, dd (12.3, 8.9) 1.42, dd (12.3, 11.3)	1.51, overlapped 1.38, dd (12.8, 11.8)	1.55, dd (11.7, 11.7) 1.43, dd (11.7, 8.5)	1.65, br dd (13.2, 8.3) 1.57, dd (13.2, 10.7)
10	2.31, d (15.3) 1.93, d (15.3)	2.24, d (15.1) 1.91, d (15.1)	2.21, br d (15.2) 2.06, br d (15.2)	2.31, d (14.8) 1.97, d (14.8)
11	4.11, d (11.3) 3.93, d (11.3)	4.11, dd (11.6, 6.5) 4.01, dd (11.6, 4.5)	4.06, d (11.3) 3.95, d (11.3)	4.03, d (12.0) 4.00, d (12.0)
12	4.01, overlapped 3.84, dd (9.2, 9.2)	4.29, dd (10.5, 7.4) 4.23, dd (10.5, 8.9)	3.73, dd (10.5, 9.0) 3.70, dd (10.5, 7.4)	4.34, dd (10.7, 10.7) 4.19, dd (10.7, 5.7)
13	0.92, d (7.0)	0.82, d (7.0)	0.87, d (7.0)	1.08, s
14	1.08, s	1.06, s	0.89, s	1.10, s
15	0.88, s	0.83, s	3.38, d (10.0) 3.40, d (10.0)	0.86, s
OAc		2.06, s		2.04, s
11-OH		1.54, dd (6.5, 4.5)		

^a^Recorded in CD_3_OD

^b^Recorded in CDCl_3_

**Table 4:** ^13^C NMR spectroscopic data of compounds **1**–**8** (150 MHz, *δ* in ppm)

No	**1**^a^	**2**^a^	**3**^b^	**4**^c^	**5**^a^	**6**^a,d^	**7**^a,d^	**8**^a^
1	133.1, C	37.8, CH	170.1, C	93.7, C	143.3, C	135.39, C	136.07, C	140.6, C
2	134.5, C	145.5, C	123.3, C	156.1, C	137.9, C	131.28, C	131.65, C	126.8, C
3	46.3, CH	139.4, C	74.4, C	52.2, CH	41.1, CH	45.15, CH	47.04, CH	38.8, CH
4	31.4, CH_2_	29.5, CH_2_	26.8, CH_2_	26.7, CH_2_	38.4, CH_2_	34.39, CH_2_	35.81, CH_2_	31.1, CH_2_
5	80.0, CH	74.7, CH	30.1, CH_2_	29.4, CH_2_	112.8, C	76.79, CH	76.48, CH	73.6, CH
6	44.4, C	41.6, CH	32.4, CH	32.1, CH	43.7, CH	45.05, CH	44.91, CH	39.7, CH
7	71.4, CH	43.6, CH	48.0, CH	51.3, CH	42.9, CH	40.64, CH	40.73, CH	41.2, CH
8	44.3, CH_2_	45.2, CH_2_	43.8, CH_2_	37.6, CH_2_	45.4, CH_2_	47.10, CH_2_	47.19, CH_2_	45.5, CH_2_
9	33.4, C	37.3, C	38.9, C	41.5, C	38.4, C	38.59, C	38.46, C	39.6, C
10	39.5, CH_2_	47.2, CH_2_	49.2, CH_2_	48.9, CH_2_	48.6, CH_2_	47.22, CH_2_	47.96, CH_2_	47.4, CH_2_
11	68.2, CH_2_	172.7, C	168.6, C	107.1, CH_2_	65.3, CH_2_	69.53, CH_2_	68.56, CH_2_	70.9, CH_2_
12	111.3, CH	173.2, C	179.8, C	106.2, CH	75.1, CH_2_	100.99, CH	104.78, CH	182.6, C
13	18.0, CH_3_	12.6, CH_3_	11.6, CH_3_	14.7, CH_3_	12.7, CH_3_	14.96, CH_3_	14.81, CH_3_	12.1, CH_3_
14	25.6, CH_3_	32.0, CH_3_	27.3, CH_3_	26.1, CH_3_	29.3, CH_3_	29.78, CH_3_	29.70, CH_3_	27.5, CH_3_
15	32.5, CH_3_	31.6, CH_3_	28.5, CH_3_	71.1, CH_2_	27.3, CH_3_	28.24, CH_3_	28.16, CH_3_	29.0, CH_3_
1′		41.3, CH_2_	41.5, CH_2_					
2′		60.4, CH_2_	61.0, CH_2_					
MeO-	56.8, CH_3_			53.6, OCH_3_				

^a^Recorded in CD_3_OD

^b^Recorded in CDCl_3_

^c^Recorded in acetone-*d*_6_

^d^Assignments were rounded to two decimal places to tell apart the close resonances

**Table 5:** ^13^C NMR spectroscopic data of compounds **9**–**15** (150 MHz, *δ* in ppm)

No	**9**^a^	**10**^b^	**11**^b^	**12**^a^	**13**^b^	**14**^a^	**15**^a^
1	41.3, CH	40.4, CH	39.1, CH	146.9, C	146.1, C	144.8, C	145.0, C
2	168.1, C	163.1, C	163.5, C	131.1, C	131.4, C	134.3, C	133.9, C
3	127.9, C	128.1, C	127.9, C	52.8, CH	40.7, CH	45.8, CH	40.3, CH
4	20.3, CH_2_	19.9, CH_2_	20.0, CH_2_	67.5, CH	21.5, CH_2_	22.2, CH_2_	25.4, CH_2_
5	43.0, CH_2_	32.1, CH_2_	33.5, CH_2_	42.6, CH_2_	31.9, CH_2_	33.2, CH_2_	42.2, CH_2_
6	75.0, C	33.4, CH	32.2, CH	33.9, CH	31.4, CH	33.3, CH	73.3, C
7	55.7, CH	48.6, CH	48.6, CH	46.6, CH	46.2, CH	47.1, CH	53.5, CH
8	42.9, CH_2_	39.78, CH_2_^c^	40.1, CH_2_	46.5, CH_2_	45.3, CH_2_	41.4, CH_2_	44.0, CH_2_
9	37.3, C	39.84, C^*c*^	41.6, C	38.2, C	37.1, C	43.7, C	37.1, C
10	45.1, CH_2_	39.0, CH_2_	39.2, CH_2_	48.7, CH_2_	47.8, CH_2_	43.8, CH_2_	48.1, CH_2_
11	72.3, CH_2_	70.5, CH_2_	70.6, CH_2_	66.1, CH_2_	66.3, CH_2_	66.2, CH_2_	66.1. CH_2_
12	177.6, C	175.4, C	175.5, C	60.2, CH_2_	63.7, CH_2_	61.8, CH_2_	63.9, CH_2_
13	21.1, CH_3_	11.5, CH_3_	11.6, CH_3_	12.9, CH_3_	11.6, CH_3_	12.1, CH_3_	19.9, CH_3_
14	31.4, CH_3_	26.9, CH_3_	26.5, CH_3_	29.0, CH_3_	28.5, CH_3_	22.8, CH_3_	29.0, CH_3_
15	31.1, CH_3_	72.2, CH_2_	71.4, CH_2_	27.3, CH_3_	26.8, CH_3_	71.6, CH_2_	27.2, CH_3_
CH_3_CO-		171.5, C			171.1, C		173.1, C
CH_3_CO-		21.2, CH_3_			21.1, CH_3_		21.0, CH_3_

^a^Recorded in CD_3_OD

^b^Recorded in CDCl_3_

^c^Assignments were rounded to two decimal places to tell apart the close resonances

The molecular formula of irpexolactin L (**13**) was assigned as C_17_H_28_O_3_ by HRESIMS (*m/z* 303.1941 [M + Na]^+^, calcd for C_17_H_28_O_3_Na, 303.1941), accounting for four degrees of unsaturation. The ^1^H and ^13^C NMR data of **13** (Tables 3, 5) showed features like those of tremulenediol A (**18**) [3], except the presence of an acetyl group (*δ*_H_ 2.06; *δ*_C_ 21.1 and 171.1). The HMBC correlation from H-12 (*δ*_H_ 4.29 and 4.23) to carbonyl of acetyl group, suggesting an acetoxy group substituted at C-12. Therefore, compound **13** was elucidated as shown in Fig. 1.

Compound **14** was obtained as a colorless oil and found to possess the molecular formula of C_15_H_26_O_3_ based on HRESIMS data (*m/z* 277.1778 [M + Na]^+^, calcd for C_15_H_26_O_3_Na, 277.1774). The ^1^H and ^13^C NMR data of **14** (Tables 3, 5) were highly similar to those of conocenol A [11]. The most significant deviation of the ^13^C NMR data between conocenol A and **14** located on the hydroxymethyl (*δ*_C_ 68.7 for conocenol A (C-15), *δ*_C_ 71.6 for **14** (C-14)) which were recorded in the same deuterium solvent (CD_3_OD), implying the only difference involved in the configuration of hydroxymethyl group attached to C-9. The crucial ROESY correlations of H-7/H-8*α*, H-15\H-8*β* helped to determine the *β* orientation of 15-CH_2_OH, while it was *α* in conocenol A when its relative configuration was drawn as same as **14**. Thus, compound **14** was established as irpexolactin M.

Irpexolactin N (**15**), a colorless oil, showed a sodium adduct ion peak at *m/z* 319.1871 [M + Na]^+^ in the HRESIMS spectrum, indicating the molecular formula of C_17_H_28_O_4_ (calcd for C_17_H_28_O_4_Na, 319.1880). The 1D NMR (Tables 3, 5) spectra showed resonances of four methyl singlets, six methylenes (two oxygenated), two methines, and five quaternary carbons (Tables 3, 5). All these data showed similarity with those of (+)-(3*S*,6*R*,7*R*)-tremulene-6,11,12-triol (**19**) [14]. The presence of an additional acetyl group (*δ*_H_ 2.04; *δ*_C_ 21.0 and 173.1) in **15** as well as the HMBC correlation from H-12 (*δ*_H_ 4.34 and 4.19) to the acetyl carbonyl indicated that the 12-OH of **15** was acetylated compared to (+)-(3*S*,6*R*,7*R*)-tremulene-6,11,12-triol (**19**). Thus, compound **15** was identified as shown in Fig. 1.

The known sesquiterpenes were identified as conocenol C (**16**) [11], tremulenolide D (**17**) [13], tremulenediol A (**18**) [3], (+)-(3*S*,6*R*,7*R*)-tremulene-6,11,12-triol (**19**) [14, 15], conocenol B (**20**) [11], (−)-(3*S*,6*S*,7*S*,10*S*)-tremulene-10,11,12-triol (**21**) [14, 15], irlactam A (**22**) [6], 11,12-dihydroxy-1-tremulen-5-one (**23**) [10], and irlactin A (**24**) [4].

**Fig. 5:**
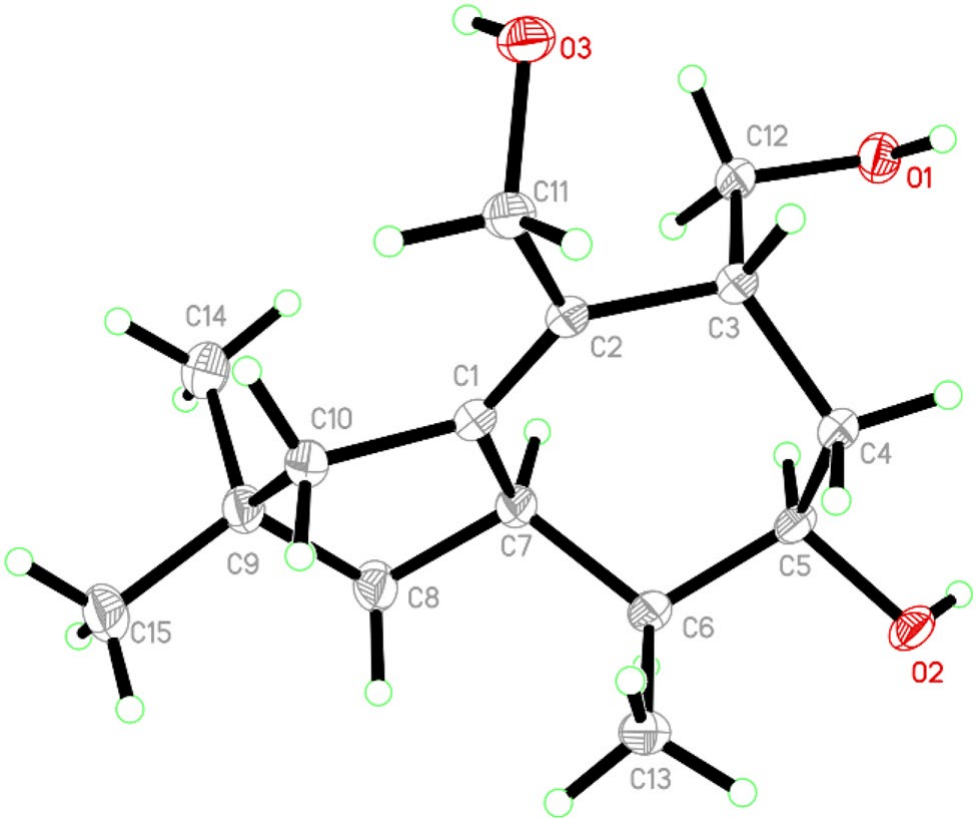
ORTEP drawing of compound **20**

The absolute configuration of conocenol B (**20**) was determined as 3*R*,5*R*,6*S*,7*R* by single-crystal X-ray diffraction analysis (Fig. 5).

### Biological Activities

Compounds **1**, **2**, **4**, **12**, and **22** were evaluated on vasorelaxant effect on KCl precontracted thoracic aorta rings. Nifedipine was used as the positive control. However, none of them showed significant vasorelaxant effect.

## Conclusions

The chemical investigation on the culture broth of the medicinal fungus *Irpex lacteus* HFG1102 facilitated the isolation of 15 previously undescribed tremulane sesquiterpenes irlactin K (**1**), and irpexolactins A–N (**2**–**15**), and nine known ones, conocenol C (**16**), tremulenolide D (**17**), tremulenediol A (**18**), (+)-(3*S*,6*R*,7*R*)-tremulene-6,11,12-triol (**19**), conocenol B (**20**), (−)-(3*S*,6*S*,7*S*,10*S*)-tremulene-10,11,12-triol (**21**), irlactam A (**22**), 11,12-dihydroxy-1-tremulen-5-one (**23**), and irlactin A (**24**). Among the all isolates, irpexolactins A (**2**) and B (**3**) possessed an unusual 1-(2-hydroxyethyl)pyrrolidine-2,5-dione moiety. This research expands the structural diversity of mushroom derived tremulane-type sesquiterpenoids.

## Experimental

### General Experimental Procedures

Optical rotations were obtained on a JASCO P-1020 digital polarimeter (Horiba, Kyoto, Japan). UV spectra were recorded on a Shimadzu UV-2401PC UV–visible recording spectrophotometer (Shimadzu, Kyoto, Japan). 1D and 2D NMR spectra were obtained on a Bruker Avance III 600 MHz spectrometer (Bruker Corporation, Karlsruhe, Germany). HRESIMS were recorded on an Agilent 6200 Q-TOF MS system (Agilent Technologies, Santa Clara, CA, USA). HREIMS were recorded on a Waters Auto-Spec Premier P776 instrument (Waters, Milford, MA, USA). Single crystal X-ray diffraction was performed on Bruker APEX II DUO and D8 QUEST diffractometers (Bruker AXS GmbH, Karlsruhe, Germany). Sephadex LH-20 (Amersham Biosciences, Uppsala, Sweden) and silica gel (Qingdao Haiyang Chemical Co., Ltd) were used for column chromatography (CC). Medium Pressure Liquid Chromatography (MPLC) was performed on a Büchi Sepacore System equipped with pump manager C-615, pump modules C-605 and fraction collector C-660 (Büchi Labortechnik AG, Flawil, Switzerland), and columns packed with Chromatorex C-18 (dimensions 450 mm × i.d. 14 mm, particle size: 40–75 μm, Fuji Silysia Chemical Ltd., Kasugai, Japan). Preparative high performance liquid chromatography (prep. HPLC) were performed on an Agilent 1260 liquid chromatography system equipped with Zorbax SB-C18 columns (particle size 5 μm, dimensions 150 mm × i.d. 9.4 mm, flow rate 7 ml·min^−1^, respectively) and a DAD detector (Agilent Technologies, Santa Clara, CA, USA).

### Fungal Material

The fungus *Irpex lacteus* was collected from Changbai Mountain Nature Reserve in 2012, and was identified by Prof. Yu-Cheng Dai (Beijing Forestry University), an expert in mushroon taxonomy. The strain of *I. lacteus* in this study was isolated from the fresh fruiting bodies and kept on potato, dextrose, and agar (PDA) culture medium. A voucher specimen (No. CGBWSHFG1102) was deposited in the Herbarium of Kunming Institute of Botany, Chinese Academy of Sciences. The culture medium to ferment this fungus consisted of glucose (5%), peptone from porcine meat (0.15%), yeast powder (0.5%), KH_2_PO_4_ (0.05%) and MgSO_4_ (0.05%). Sixty Erlenmeyer flasks (500 ml) each containing 350 ml of above-mentioned culture medium were inoculated with *I. lacteus* strains, respectively. Fermentation were carried out on rotatory shakers at 25 °C and 150 rpm for 25 days in dark environment.

### Extraction and Isolation

The culture broth (20 L) of *I. lacteus* HFG1102 was filtered and concentrated to 3 L followed by partitioned between EtOAc and water for four times to give an EtOAc layer. Meanwhile, the mycelia were extracted by EtOH (95%) for three times. The EtOAc layer together with the mycelium extract were concentrated under reduced pressure to afford a crude extract (16.0 g). This residue was separated by MPLC (ODS column) with MeOH/H_2_O (from 0:100 to 100:0) to give sixteen main fractions (A–P).

Subfraction E was fractionated by Sephadex LH-20 CC (MeOH) to afford five subfractions E1–E5. Compounds **12** (1.5 mg), **13** (2.4 mg), **14** (2.0 mg) were purified from E1 to E5, respectively, by Sephadex LH-20 CC using acetone as a mobile phase. Subfraction F was fractionated and purified by repeatedly Sephadex LH-20 (MeOH or acetone) to give compounds **16** (1.2 mg), **17** (1.8 mg), **19** (3.0 mg), **20** (2.5 mg).

Subfraction G was separated by Sephadex LH-20 (MeOH) to give four subfractions G1–G4. G4 was further separated by Sephadex LH-20 (acetone) to give three subfractions G4a–G4c. Compounds **10** (0.9 mg) and **15** (1.0 mg) were purified from G4a and G4b by prep-HPLC, respectively (**10**: MeCN/H_2_O, 30:70–54:46, 7 mL min^−1^, t_R_ = 18.5 min; **15**: MeCN/H_2_O, 25:75–45:55, 7 mL min^−1^, t_R_ = 19.5 min). Subfraction G4c was purified by prep-HPLC (MeCN/H_2_O, 28:72–53:47, 7 mL min^−1^) to give compounds **5/23** (t_R_ = 5.0 min, 1.2 mg), **6**/**7** (t_R_ = 6.5 min, 2.2 mg).

Subfraction H was separated by Sephadex LH-20 (MeOH) to give two subfractions H1 and H2. H2 was further separated by Sephadex LH-20 (acetone) to give three subfractions H2a–H2c. H2b was separated by silica gel CC to give seven subfractions H2b1–H2b7. Subfraction H2b2 was purified by prep-HPLC to yield compound **11** (MeCN/H_2_O, 18:82–38:62, 7 mL min^−1^, t_R_ = 17.5 min, 2.2 mg). Subfraction H2b7 was purified by prep-HPLC (MeCN/H_2_O, 23:77–43:57, 7 mL min^−1^) to yield compounds **2** (t_R_ = 10.5 min, 1.8 mg), **22** (t_R_ = 14.8 min, 2.3 mg), and **3** (t_R_ = 18.2 min, 1.5 mg). and **21** (2.5 mg).

Subfraction I was separated by Sephadex LH-20 (MeOH) to give two subfractions I1 and I2. Subfraction I1 was further separated by Sephadex LH-20 (acetone) and prep-HPLC (MeCN/H_2_O, 28:72–48:52, 7 mL min^−1^) to give compound **8** (t_R_ = 11.5 min, 3.2 mg). Subfraction I2 was further purified by prep-HPLC (MeCN/H_2_O, 30:70–50:50, 7 mL min^−1^) to give compound **4** (t_R_ = 14.5 min, 3.0 mg).

Subfraction J was separated by Sephadex LH-20 (MeOH) and further purified by prep-HPLC (MeCN/H_2_O, 18:82–38:62, 7 mL min^−1^) to afford compound **9** (t_R_ = 13.3 min, 2.1 mg).

Subfraction K was separated by Sephadex LH-20 (MeOH) to give two main subfractions K1 and K2. Subfraction K1 was purified by prep-HPLC (MeCN/H_2_O, 7:93–27:73, 7 ml min^−1^) to afford compound **18** (t_R_ = 14.3 min, 1.1 mg) and **24** (t_R_ = 16.0 mg, 1.5 mg). Subfraction K2 was purified by prep-HPLC (MeCN/H_2_O, 13:87–33:67, 7 mL min^−1^) to afford compound **1** (t_R_ = 18.5 min, 0.9 mg).

### Spectroscopic Data of Compounds

#### Irlactin K (1)

Colorless needles; UV (MeOH) λ_max_ nm (log *ε*): 205 (2.54); ^1^H NMR (600 MHz, CDCl_3_) data, see Table 1; ^13^C NMR (150 MHz, CDCl_3_) data, see Table 4.

#### Irpexolactin A (2)

Pale-yellow oil; [*α*]_D_^26^ + 79.2 (*c* 0.07, MeOH). UV (MeOH) λ_max_ nm (log *ε*): 200 (3.69), 231 (3.96); IR (KBr) ν_max_ 3426, 2927, 2859, 1701, 1632, 1406, 1030 cm^–1^; ^1^H NMR (600 MHz, CDCl_3_) data, see Table 1; ^13^C NMR (150 MHz, CDCl_3_) data, see Table 4; HRESIMS *m/z* 330.1685 [M + Na]^+^ (calcd for C_17_H_25_NO_4_Na, 330.1676).

#### Irpexolactin B (3)

Colorless oil; [*α*]_D_^26^ + 42.5 (*c* 0.10, MeOH). ^1^H NMR (600 MHz, CDCl_3_) data, see Table 1; ^13^C NMR (150 MHz, CDCl_3_) data, see Table 4; HRESIMS *m/z* 330.1687 [M + Na]^+^ (calcd for C_17_H_26_NO_4_Na, 330.1676).

#### Irpexolactin C (4)

Colorless oil; [*α*]_D_^26^ –95.9 (*c* 0.07, MeOH). UV (MeOH) λ_max_ nm (log *ε*): 204 (3.64); IR (KBr) ν_max_ 3428, 2926, 2858, 1632, 1384, 1031 cm^–1^; ^1^H NMR (600 MHz, CDCl_3_) data, see Table 1; ^13^C NMR (150 MHz, CDCl_3_) data, see Table 4; HRESIMS *m/z* 289.1774 [M + Na]^+^ (calcd for C_16_H_26_O_3_Na, 289.1774).

#### Irpexolactin D (5) and 23 Mixture

Colorless oil; UV (MeOH) λ_max_ nm (log *ε*): 206 (4.16), 244 (3.08); IR (KBr) ν_max_ 3426, 2951, 2932, 2870, 1691, 1631, 1462, 1383, 1027 cm^–1^; ^1^H NMR (600 MHz, CDCl_3_) data, see Table 1; ^13^C NMR (150 MHz, CDCl_3_) data, see Table 4; HRESIMS *m/z* 275.1624 [M + Na]^+^ (calcd for C_15_H_24_O_3_Na, 275.1618).

#### Irpexolactins E&F (6&7)

Yellow oil; UV (MeOH) λ_max_ nm (log *ε*): 206 (3.66), 249 (3.36); IR (KBr) ν_max_ 3426, 2953, 2928, 2861, 1632, 1385, 1029 cm^–1^; ^1^H NMR (600 MHz, CDCl_3_) data, see Table 2; ^13^C NMR (150 MHz, CDCl_3_) data, see Table 4; HRESIMS *m/z* 275.1621 [M + Na]^+^ (calcd for C_15_H_24_O_3_Na, 275.1618).

#### Irpexolactin G (8)

Colorless oil; [*α*]_D_^26^ + 17.1 (*c* 0.06, MeOH). UV (MeOH) λ_max_ nm (log *ε*): 207 (3.66); IR (KBr) ν_max_ 3421, 2953, 2928, 2869, 1755, 1629, 1381, 1171, 1029 cm^–1^; ^1^H NMR (600 MHz, CDCl_3_) data, see Table 2; ^13^C NMR (150 MHz, CDCl_3_) data, see Table 4; HRESIMS *m/z* 273.1468 [M + Na]^+^ (calcd for C_15_H_22_O_3_Na, 273.1461).

#### Irpexolactin H (9)

Colorless oil; [*α*]_D_^26^ + 5.7 (*c* 0.14, MeOH). UV (MeOH) λ_max_ nm (log *ε*): 219 (3.71), 240.0 (3.27); IR (KBr) ν_max_ 3424, 2928, 2859, 1726, 1631, 1384, 1029 cm^–1^; ^1^H NMR (600 MHz, CDCl_3_) data, see Table 2; ^13^C NMR (150 MHz, CDCl_3_) data, see Table 5; HRESIMS *m/z* 273.1465 [M + Na]^+^ (calcd for C_15_H_22_O_3_Na, 273.1461).

#### Irpexolactin I (10)

Colorless oil; [*α*]_D_^26^ + 2.7 (*c* 0.10, MeOH). UV (MeOH) λ_max_ nm (log *ε*): 210 (3.15); IR (KBr) ν_max_ 3427, 2926, 2856, 1714, 1633, 1386, 1031 cm^–1^; ^1^H NMR (600 MHz, CDCl_3_) data, see Table 2; ^13^C NMR (150 MHz, CDCl_3_) data, see Table 5; HRESIMS *m/z* 315.1560 [M + Na]^+^ (calcd for C_17_H_24_O_4_Na, 315.1567).

#### Irpexolactin J (11)

Colorless oil; [*α*]_D_^25^ + 17.1 (*c* 0.08, MeOH). UV (MeOH) λ_max_ nm (log *ε*): 219 (3.74); IR (KBr) ν_max_ 3426, 2928, 2869, 1732, 1633, 1452, 1389, 1031 cm^–1^; ^1^H NMR (600 MHz, CDCl_3_) data, see Table 2; ^13^C NMR (150 MHz, CDCl_3_) data, see Table 5; HRESIMS *m/z* 273.1467 [M + Na]^+^ (calcd for C_15_H_22_O_3_Na, 273.1461).

#### Irpexolactin K (12)

Colorless oil; [*α*]_D_^26^ + 23.1 (*c* 0.08, MeOH). UV (MeOH) λ_max_ nm (log *ε*): 208 (3.83); IR (KBr) ν_max_ 3427, 2927, 2857, 1633, 1391, 1028 cm^–1^; ^1^H NMR (600 MHz, CDCl_3_) data, see Table 3; ^13^C NMR (150 MHz, CDCl_3_) data, see Table 5; HRESIMS *m/z* 277.1781 [M + Na]^+^ (calcd for C_15_H_26_O_3_Na, 277.1774).

#### Irpexolactin L (13)

Colorless oil; [*α*]_D_^25^ + 78.3 (*c* 0.10, MeOH). UV (MeOH) λ_max_ nm (log *ε*): 210 (3.73); IR (KBr) ν_max_ 3438, 2925, 2856, 1631, 1385, 1030 cm^–1^; ^1^H NMR (600 MHz, CDCl_3_) data, see Table 3; ^13^C NMR (150 MHz, CDCl_3_) data, see Table 5; HRESIMS *m/z* 303.1941 [M + Na]^+^ (calcd for C_17_H_28_O_3_Na, 303.1941).

#### Irpexolactin M (14)

Colorless oil; [*α*]_D_^26^ + 96.1 (*c* 0.10, MeOH). UV (MeOH) λ_max_ nm (log *ε*): 207 (4.14), 252 (2.53); IR (KBr) ν_max_ 3426, 2927, 2866, 1633, 1385, 1028 cm^–1^; ^1^H NMR (600 MHz, CDCl_3_) data, see Table 3; ^13^C NMR (150 MHz, CDCl_3_) data, see Table 5; HRESIMS *m/z* 277.1778 [M + Na]^+^ (calcd for C_15_H_26_O_3_Na, 277.1774).

#### Irpexolactin N (15)

Colorless oil; [*α*]_D_^25^ + 21.2 (*c* 0.04, MeOH). UV (MeOH) λ_max_ nm (log *ε*): 208 (3.76), 250 (3.06); IR (KBr) ν_max_ 3422, 2928, 2859, 1721, 1630, 1457, 1384, 1266, 1093, 1031 cm^–1^; ^1^H NMR (600 MHz, CDCl_3_) data, see Table 3; ^13^C NMR (150 MHz, CDCl_3_) data, see Table 5; HRESIMS *m/z* 319.1871 [M + Na]^+^ (calcd for C_17_H_28_O_4_Na, 319.1880).

#### Single-Crystal X-ray Diffraction Data of 1

Colorless crystals of **1** were obtained by crystallization from MeOH/H_2_O/petroleum ether. The crystal data were obtained from an APEX II DUO spectrophotometer (Bruker AXS GmbH, Karlsruhe, Germany) using graphite-monochromated Cu K*a* radiation (*λ* = 1.54178 Å). The structures of **1** were solved by the direct method employing the SHELXS-97 program and then refined with full-matrix least-squares difference Fourier techniques. Crystallographic data of compound **1** was deposited to the Cambridge Crystallographic Data Centre (No. CCDC 1823228). These data can be obtained free of charge via https://www.ccdc.cam.ac.uk/conts/retrieving.html. Crystal data for Cu_**1**_0m: C_16_H_26_O_4_, *M* = 282.37, *a* = 5.8192(2) Å, *b* = 9.6527(4) Å, *c* = 13.5674(6) Å, *α* = 90°, *β* = 93.526(2)°, *γ* = 90°, *V* = 760.65(5) Å^3^, *T* = 100(2) K, space group *P*21, *Z* = 2, *μ*(CuKα) = 0.702 mm^−1^, 6956 reflections measured, 2415 independent reflections (*R*_*int*_ = 0.0287). The final *R*_*1*_ values were 0.0300 (*I* > 2*σ*(*I*)). The final *wR*(*F*^2^) values were 0.0797 (*I* > 2*σ*(*I*)). The final *R*_*1*_ values were 0.0302 (all data). The final *wR*(*F*^2^) values were 0.0799 (all data). The goodness of fit on *F*^2^ was 1.070. Flack parameter = 0.15(6).

#### Single-Crystal X-ray Diffraction Data of 20

A light colorless platelet-like of C_15_H_26_O_3_, *M* = 254.36, approximate dimensions 0.095 × 0.116 × 0.263 mm^3^, was used for the X-ray crystallographic analysis on the BRUKER D8 QUEST diffractometer. The integration of the data using a monoclinic unit cell yielded a total of 12,363 reflections to a maximum *θ* angle of 79.29° (0.78 Å resolution), of which 3012 were independent (average redundancy 4.105, completeness = 98.1%, R_int_ = 5.36%, R_sig_ = 5.40%) and 2848 (94.56%) were greater than 2σ(F^2^). The final cell constants of a = 6.1670(3) Å, b = 7.7139(4) Å, c = 15.5645(8) Å, α = 90.00°, β = 93.047(2)°, γ = 90.00°, V = 739.38(6) Å^3^, T = 150.(2) K. Data were corrected for absorption effects using the Multi-Scan method (SADABS). The structure was solved and refined using the Bruker SHELXTL Software Package, using the space group P 1 21 1, with Z = 2, μ(CuKα) = 1.54178. The final anisotropic full-matrix least-squares refinement on F^2^ with 174 variables converged at R1 = 3.54%, for the observed data and *ωR*^2^ = 8.84% for all data. The goodness-of-fit was 1.009. The absolute configuration was determined by the Flack parameter = 0.01(9), which was determined using 1165 quotients [(I+) − (I−)]/[(I+) + (I−)]. Crystallographic data of compound **20** was deposited to the Cambridge Crystallographic Data Centre (No. CCDC 1977047). These data can be obtained free of charge via https://www.ccdc.cam.ac.uk/conts/retrieving.html.

### Vasorelaxant Effect Assay

#### Animals

Adult male Wistar rats (250–300 g) were kept in an animal room with a constant temperature of 22 ± 2 ℃, a humidity of 60 ± 5% and had free access to food and water. Experiments were performed in accordance with the guidelines of the National Institutes of Health Guide for the Care and Use of Laboratory Animals. All experimental procedures were approved by the Research Ethics Committee of the Kunming Institute of Botany, Chinese Academy of Sciences.

#### Methods

Vasorelaxant effects of 100 µmol/L of the compounds was evaluated on endothelium-intact thoracic aorta rings precontracted with KCl. Rat aortic rings were prepared according to that described [16, 17]. Nifedipine, a calcium channel blocker, was used as the positive control. Aortic rings were mounted on stainless steel hooks in organ baths containing 37 ℃ Krebs solution continuously bubbled with 95% O_2_ and 5% CO_2_, then equilibrated for 60 min under a resting tension of 1.5 g. After equilibration, the vessels were exposed to 1 μmol/l phenylephrine (PE), followed by 10 μmol/l acetylcholine (Ach) to check functional endothelial integrity, more than 80% relaxation of the rings was considered to be an endothelium-intact ring. Endothelium-intact rings precontracted with 60 mmol/l KCl were treated with different compounds, DMSO or nifedipine for 30 min or 60 min, the changes in tension of aortic rings were recorded. The vasorelaxant effect was calculated as a percentage of the relaxation in response to KCl on the aortic rings. Data were presented as mean ± SD and evaluated by one-way ANOVA followed by Bonferroni's test using SPSS 19.0. P < 0.05 was regarded to be statistically significant.

## Electronic supplementary material

Below is the link to the electronic supplementary material.

Supplementary file1 (DOCX 26863 kb)
