# Supplementary material for: Irlactane and Tremulane Sesquiterpenes from the Cultures of the Medicinal Fungus Irpex lacteus HFG1102
Source: Nat Prod Bioprospect. 2020 Apr 10;10(2):89–100. doi: 10.1007/s13659-020-00239-z (PMC7176803; doi:10.1007/s13659-020-00239-z)

**Supplementary data for**

**Irlactane and Tremulane Sesquiterpenoids from the Cultures of the Medicinal Fungus *Irpex lacteus* HFG1102**

He-Ping Chen,^a^ Xu Ji,^b^ Zheng-Hui Li,^a^ Tao Feng,^a^ Ji-Kai Liu^a,^*

*^a^School of Pharmaceutical Sciences, South-Central University for Nationalities, Wuhan 430074, People’s Republic of China*

*^b^School of Chemical Science and Technology, Yunnan University, Kunming 650091, People’s Republic of China*

**Corresponding authors**

*E-mails: [jkliu@mail.kib.ac.cn](mailto:jkliu@mail.kib.ac.cn); liujikai@mail.scuec.edu.cn (J.-K. Liu)

**Contents**

[Figure 1S. ^1^H NMR spectrum of **1** (600 MHz, CDCl_3_). 4](#_Toc30628278)

[Figure 2S. ^13^C NMR and DEPT spectra of **1** (150 MHz, CDCl_3_). 4](#_Toc30628279)

[Figure 3S. HSQC spectrum of **1**. 5](#_Toc30628280)

[Figure 4S. ^1^H-^1^H COSY spectrum of **1**. 5](#_Toc30628281)

[Figure 5S. HMBC spectrum of **1**. 6](#_Toc30628282)

[Figure 6S. ROESY spectrum of **1**. 6](#_Toc30628283)

[Figure 7S. ^1^H NMR spectrum of **2** (600 MHz, CDCl_3_). 7](#_Toc30628284)

[Figure 8S. ^13^C NMR and DEPT spectra of **2** (150 MHz, CDCl_3_). 7](#_Toc30628285)

[Figure 9S. HSQC spectrum of **2**. 8](#_Toc30628286)

[Figure 10S. ^1^H-^1^H COSY spectrum of **2**. 8](#_Toc30628287)

[Figure 11S. HMBC spectrum of **2**. 9](#_Toc30628288)

[Figure 12S. ROESY spectrum of **2**. 9](#_Toc30628289)

[Figure 13S. (+)-HRESIMS report of **2**. 10](#_Toc30628290)

[Figure 14S. ^1^H NMR spectrum of **3** (600 MHz, CDCl_3_). 11](#_Toc30628291)

[Figure 15S. ^13^C NMR spectrum of **3** (150 MHz, CDCl_3_). 11](#_Toc30628292)

[Figure 16S. HSQC spectrum of **3**. 12](#_Toc30628293)

[Figure 17S. ^1^H-^1^H COSY spectrum of **3**. 12](#_Toc30628294)

[Figure 18S. HMBC spectrum of **3**. 13](#_Toc30628295)

[Figure 19S. ROESY spectrum of **3**. 13](#_Toc30628296)

[Figure 20S. (+)-HRESIMS report of **3**. 14](#_Toc30628297)

[Figure 21S. ^1^H NMR spectrum of **4** (600 MHz, CDCl_3_). 15](#_Toc30628298)

[Figure 22S. ^13^C NMR and DEPT spectra of **4** (150 MHz, CDCl_3_). 15](#_Toc30628299)

[Figure 23S. HSQC spectrum of **4**. 16](#_Toc30628300)

[Figure 24S. ^1^H-^1^H COSY spectrum of **4**. 16](#_Toc30628301)

[Figure 25S. HMBC spectrum of **4**. 17](#_Toc30628302)

[Figure 26S. ROESY spectrum of **4**. 17](#_Toc30628303)

[Figure 27S. (+)-HRESIMS report of **4**. 18](#_Toc30628304)

[Figure 28S. ^1^H NMR spectrum of **5** (600 MHz, CDCl_3_). 19](#_Toc30628305)

[Figure 29S. ^13^C NMR and DEPT spectra of **5** (150 MHz, CDCl_3_). 19](#_Toc30628306)

[Figure 30S. HSQC spectrum of **5**. 20](#_Toc30628307)

[Figure 31S. ^1^H-^1^H COSY spectrum of **5**. 20](#_Toc30628308)

[Figure 32S. HMBC spectrum of **5**. 21](#_Toc30628309)

[Figure 33S. ROESY spectrum of **5**. 21](#_Toc30628310)

[Figure 34S. (+)-HRESIMS report of **5**. 22](#_Toc30628311)

[Figure 35S. ^1^H NMR spectrum of **6/7** (600 MHz, CDCl_3_). 23](#_Toc30628312)

[Figure 36S. ^13^C NMR and DEPT spectra of **6/7** (150 MHz, CDCl_3_). 23](#_Toc30628313)

[Figure 37S. HSQC spectrum of **6/7**. 24](#_Toc30628314)

[Figure 38S. ^1^H-^1^H COSY spectrum of **6/7**. 24](#_Toc30628315)

[Figure 39S. HMBC spectrum of **6/7**. 25](#_Toc30628316)

[Figure 40S. ROESY spectrum of **6/7**. 25](#_Toc30628317)

[Figure 41S. (+)-HRESIMS report of **6/7**. 26](#_Toc30628318)

[Figure 42S. ^1^H NMR spectrum of **8** (600 MHz, CDCl_3_). 27](#_Toc30628319)

[Figure 43S. ^13^C NMR and DEPT spectra of **8** (150 MHz, CDCl_3_). 27](#_Toc30628320)

[Figure 44S. HSQC spectrum of **8**. 28](#_Toc30628321)

[Figure 45S. HMBC spectrum of **8**. 28](#_Toc30628322)

[Figure 46S. ROESY spectrum of **8**. 29](#_Toc30628323)

[Figure 47S. (+)-HRESIMS report of **8**. 30](#_Toc30628324)

[Figure 48S. ^1^H NMR spectrum of **9** (600 MHz, CDCl_3_). 31](#_Toc30628325)

[Figure 49S. ^13^C NMR and DEPT spectra of **9** (150 MHz, CDCl_3_). 31](#_Toc30628326)

[Figure 50S. HSQC spectrum of **9**. 32](#_Toc30628327)

[Figure 51S. ^1^H-^1^H COSY spectrum of **9**. 32](#_Toc30628328)

[Figure 52S. HMBC spectrum of **9**. 33](#_Toc30628329)

[Figure 53S. ROESY spectrum of **9**. 33](#_Toc30628330)

[Figure 54S. (+)-HRESIMS report of **9**. 34](#_Toc30628331)

[Figure 55S. ^1^H NMR spectrum of **10** (600 MHz, CDCl_3_). 35](#_Toc30628332)

[Figure 56S. ^13^C NMR spectrum of **10** (150 MHz, CDCl_3_). 35](#_Toc30628333)

[Figure 57S. HMBC spectrum of **10**. 36](#_Toc30628334)

[Figure 58S. ROESY spectrum of **10**. 36](#_Toc30628335)

[Figure 59S. (+)-HRESIMS report of **10**. 37](#_Toc30628336)

[Figure 60S. ^1^H NMR spectrum of **11** (600 MHz, CDCl_3_). 38](#_Toc30628337)

[Figure 61S. ^13^C NMR and DEPT spectra of **11** (150 MHz, CDCl_3_). 38](#_Toc30628338)

[Figure 62S. HSQC spectrum of **11**. 39](#_Toc30628339)

[Figure 63S. ^1^H-^1^H COSY spectrum of **11**. 39](#_Toc30628340)

[Figure 64S. HMBC spectrum of **11**. 40](#_Toc30628341)

[Figure 65S. ROESY spectrum of **11**. 40](#_Toc30628342)

[Figure 66S. (+)-HRESIMS report of **11**. 41](#_Toc30628343)

[Figure 67S. ^1^H NMR spectrum of **12** (600 MHz, CDCl_3_). 42](#_Toc30628344)

[Figure 68S. ^13^C NMR and DEPT spectra of **12** (150 MHz, CDCl_3_). 42](#_Toc30628345)

[Figure 69S. HSQC spectrum of **12**. 43](#_Toc30628346)

[Figure 70S. ^1^H-^1^H COSY spectrum of **12**. 43](#_Toc30628347)

[Figure 71S. HMBC spectrum of **12**. 44](#_Toc30628348)

[Figure 72S. ROESY spectrum of **12**. 44](#_Toc30628349)

[Figure 73S. (+)-HRESIMS report of **12**. 45](#_Toc30628350)

[Figure 74S. ^1^H NMR spectrum of **13** (600 MHz, CDCl_3_). 46](#_Toc30628351)

[Figure 75S. ^13^C NMR and DEPT spectra of **13** (150 MHz, CDCl_3_). 46](#_Toc30628352)

[Figure 76S. HSQC spectrum of **13**. 47](#_Toc30628353)

[Figure 77S. ^1^H-^1^H COSY spectrum of **13**. 47](#_Toc30628354)

[Figure 78S. HMBC spectrum of **13**. 48](#_Toc30628355)

[Figure 79S. ROESY spectrum of **13**. 48](#_Toc30628356)

[Figure 80S. (+)-HRESIMS report of **13**. 49](#_Toc30628357)

[Figure 84S. ^1^H NMR spectrum of **14** (600 MHz, CDCl_3_). 50](#_Toc30628358)

[Figure 85S. ^13^C NMR and DEPT spectra of **14** (150 MHz, CDCl_3_). 50](#_Toc30628359)

[Figure 86S. HSQC spectrum of **14**. 51](#_Toc30628360)

[Figure 87S. ^1^H-^1^H COSY spectrum of **14**. 51](#_Toc30628361)

[Figure 88S. HMBC spectrum of **14**. 52](#_Toc30628362)

[Figure 89S. ROESY spectrum of **14**. 52](#_Toc30628363)

[Figure 90S. (+)-HRESIMS report of **14**. 53](#_Toc30628364)

[Figure 81S. ^1^H NMR spectrum of **15** (600 MHz, CDCl_3_). 54](#_Toc30628365)

[Figure 82S. ^13^C NMR spectrum of **15** (150 MHz, CDCl_3_). 54](#_Toc30628366)

[Figure 83S. HSQC spectrum of **15**. 55](#_Toc30628367)

[Figure 84S. ^1^H-^1^H COSY spectrum of **15**. 55](#_Toc30628368)

[Figure 85S. HMBC spectrum of **15**. 56](#_Toc30628369)

[Figure 86S. ROESY spectrum of **15**. 56](#_Toc30628370)

[Figure 87S. (+)-HRESIMS report of **15**. 57](#_Toc30628371)

# Figure 1S. ^1^H NMR spectrum of **1** (600 MHz, CDCl_3_).

# Figure 2S. ^13^C NMR and DEPT spectra of **1** (150 MHz, CDCl_3_).

# Figure 3S. HSQC spectrum of **1**.

# Figure 4S. ^1^H-^1^H COSY spectrum of **1**.

# Figure 5S. HMBC spectrum of **1**.

# Figure 6S. ROESY spectrum of **1**.

# Figure 7S. ^1^H NMR spectrum of **2** (600 MHz, CDCl_3_).

# Figure 8S. ^13^C NMR and DEPT spectra of **2** (150 MHz, CDCl_3_).

# Figure 9S. HSQC spectrum of **2**.

# Figure 10S. ^1^H-^1^H COSY spectrum of **2**.

# Figure 11S. HMBC spectrum of **2**.

# Figure 12S. ROESY spectrum of **2**.

# Figure 13S. (+)-HRESIMS report of **2**.


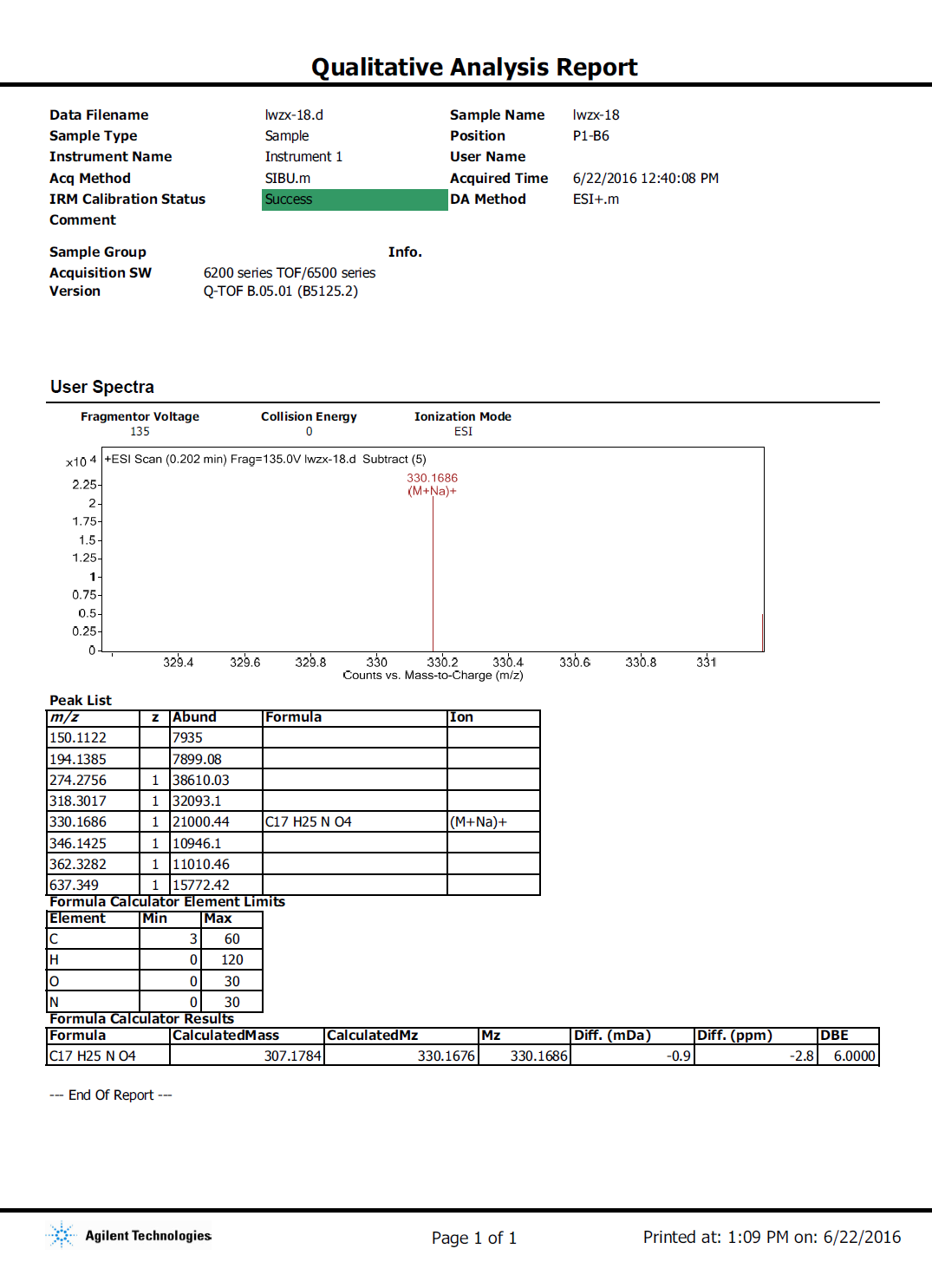


# Figure 14S. ^1^H NMR spectrum of **3** (600 MHz, CDCl_3_).

# Figure 15S. ^13^C NMR spectrum of **3** (150 MHz, CDCl_3_).

# Figure 16S. HSQC spectrum of **3**.

# Figure 17S. ^1^H-^1^H COSY spectrum of **3**.

# Figure 18S. HMBC spectrum of **3**.

# Figure 19S. ROESY spectrum of **3**.

# Figure 20S. (+)-HRESIMS report of **3**.


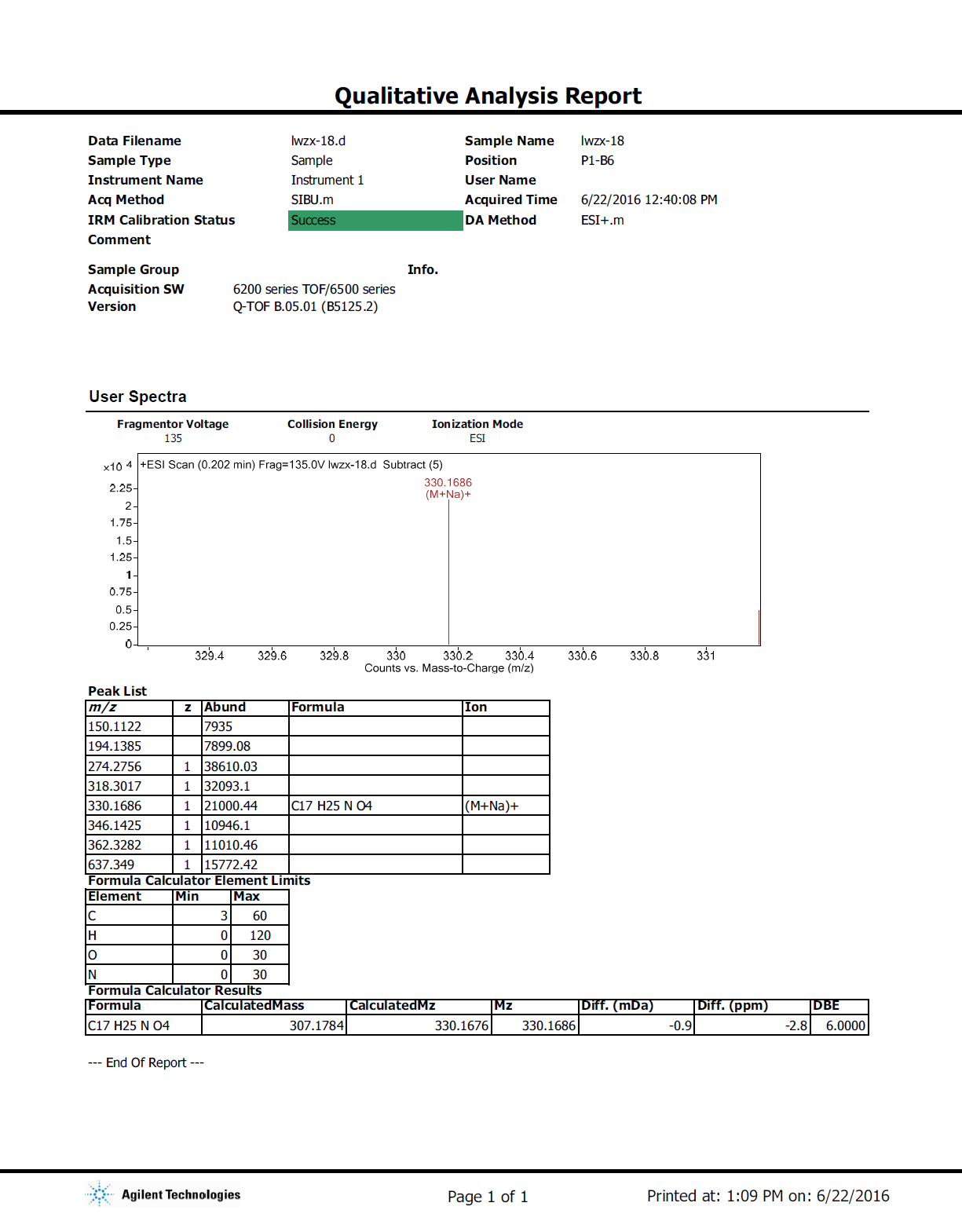


# Figure 21S. ^1^H NMR spectrum of **4** (600 MHz, CDCl_3_).

# Figure 22S. ^13^C NMR and DEPT spectra of **4** (150 MHz, CDCl_3_).

# Figure 23S. HSQC spectrum of **4**.

# Figure 24S. ^1^H-^1^H COSY spectrum of **4**.

# Figure 25S. HMBC spectrum of **4**.

# Figure 26S. ROESY spectrum of **4**.

# Figure 27S. (+)-HRESIMS report of **4**.


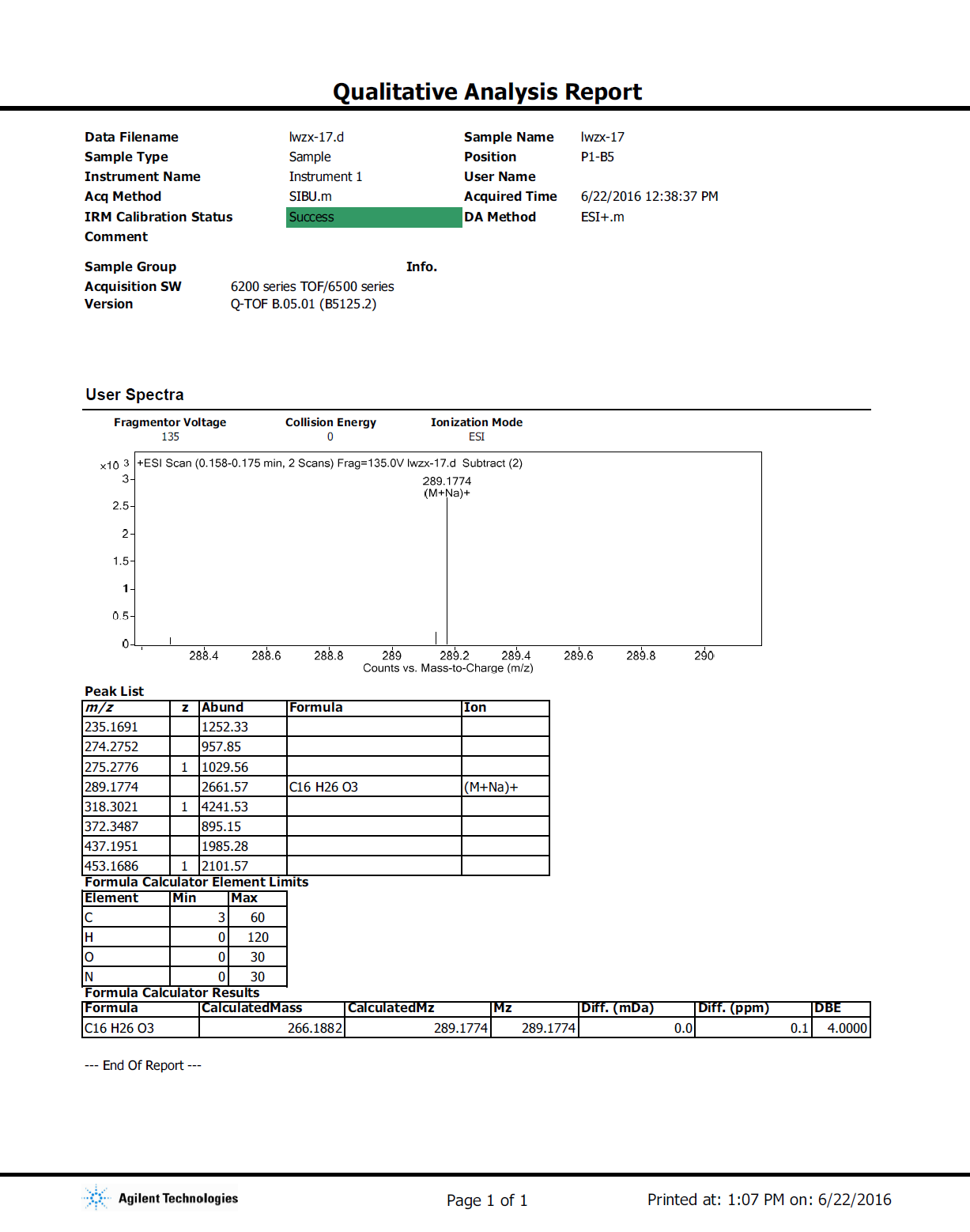


# Figure 28S. ^1^H NMR spectrum of **5** (600 MHz, CDCl_3_).

# Figure 29S. ^13^C NMR and DEPT spectra of **5** (150 MHz, CDCl_3_).

# Figure 30S. HSQC spectrum of **5**.

# Figure 31S. ^1^H-^1^H COSY spectrum of **5**.

# Figure 32S. HMBC spectrum of **5**.

# Figure 33S. ROESY spectrum of **5**.

# Figure 34S. (+)-HRESIMS report of **5**.


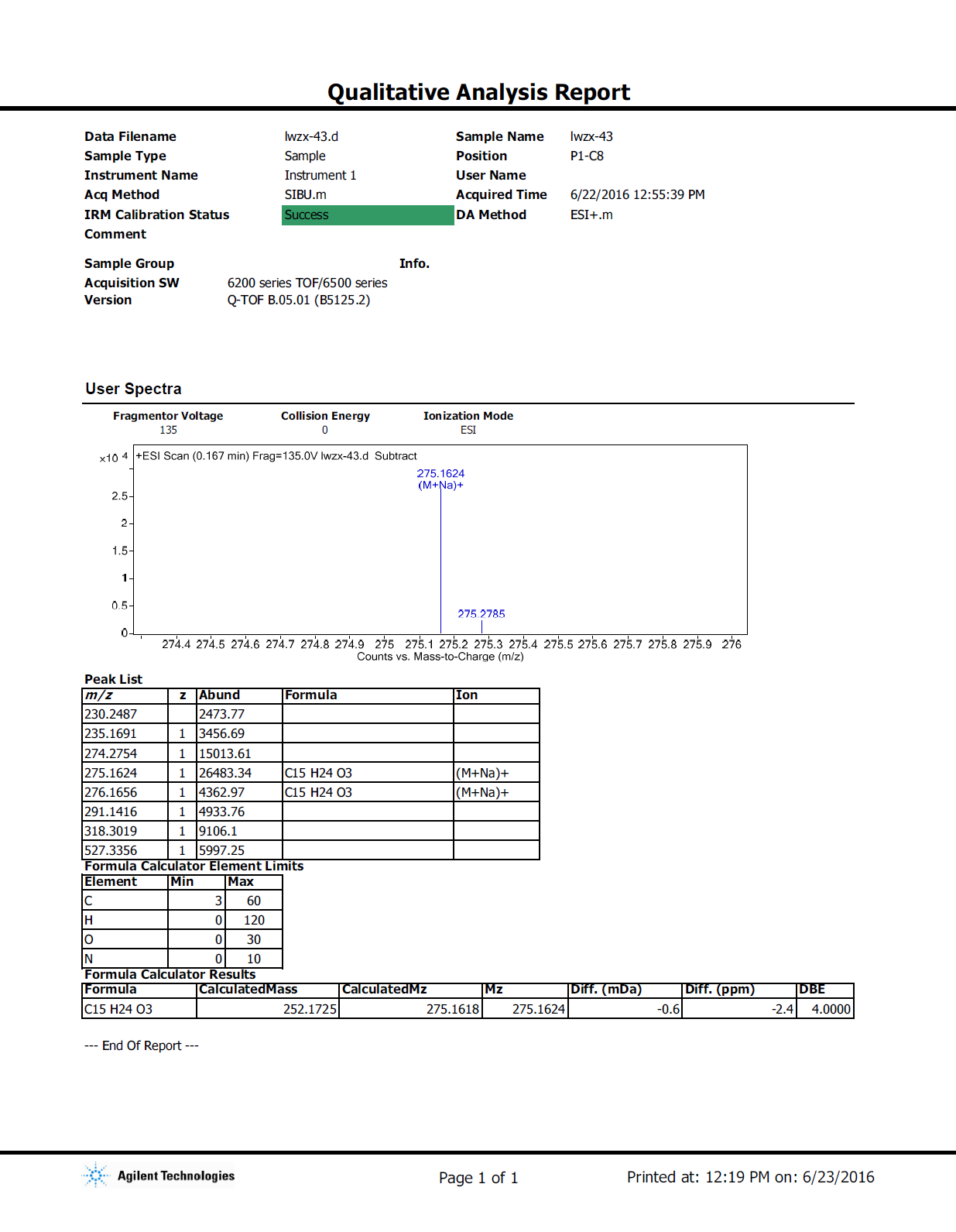


# Figure 35S. ^1^H NMR spectrum of **6/7** (600 MHz, CDCl_3_).

# Figure 36S. ^13^C NMR and DEPT spectra of **6/7** (150 MHz, CDCl_3_).

# Figure 37S. HSQC spectrum of **6/7**.

# Figure 38S. ^1^H-^1^H COSY spectrum of **6/7**.

# Figure 39S. HMBC spectrum of **6/7**.

# Figure 40S. ROESY spectrum of **6/7**.

# Figure 41S. (+)-HRESIMS report of **6/7**.


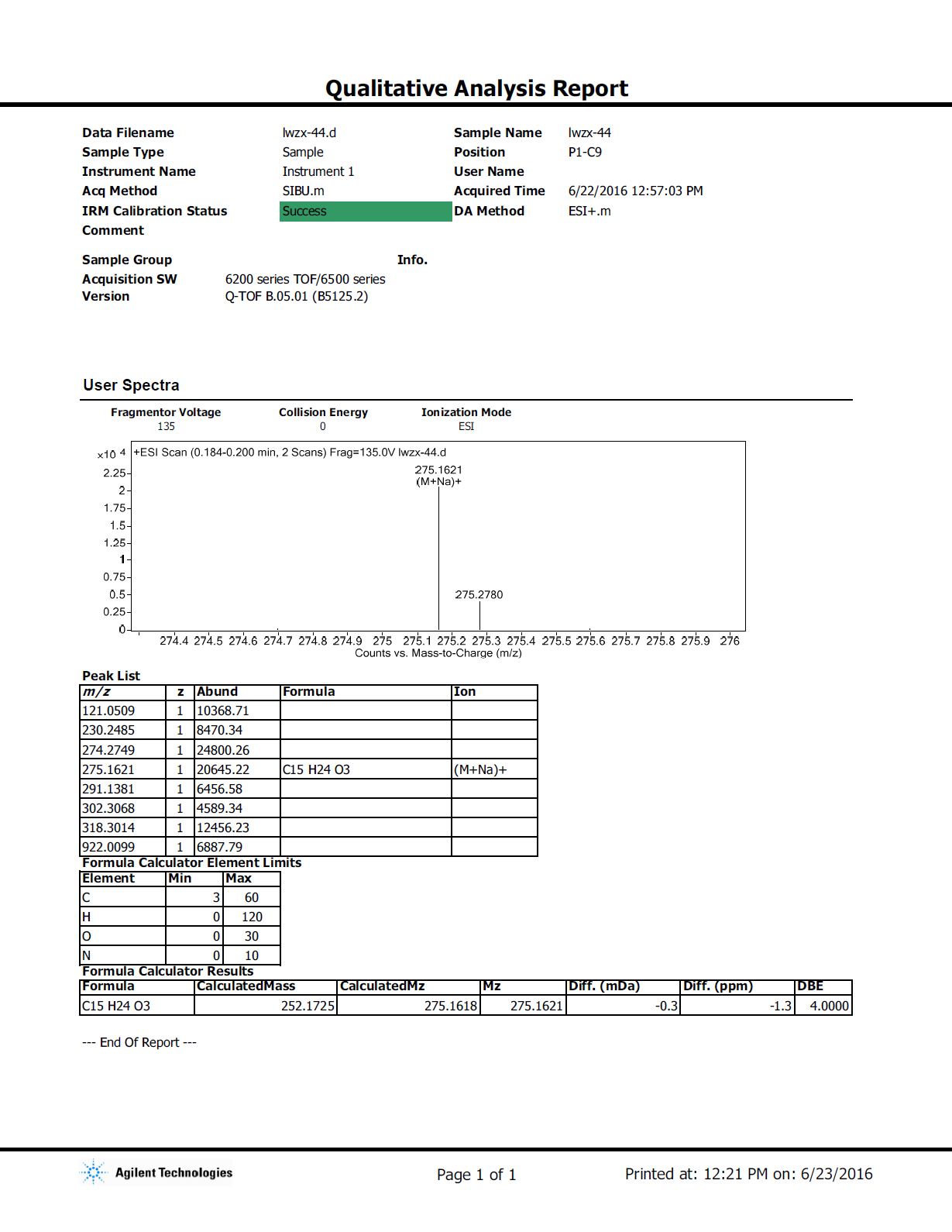


# Figure 42S. ^1^H NMR spectrum of **8** (600 MHz, CDCl_3_).

# Figure 43S. ^13^C NMR and DEPT spectra of **8** (150 MHz, CDCl_3_).

# Figure 44S. HSQC spectrum of **8**.

# Figure 45S. HMBC spectrum of **8**.

# Figure 46S. ROESY spectrum of **8**.

# Figure 47S. (+)-HRESIMS report of **8**.


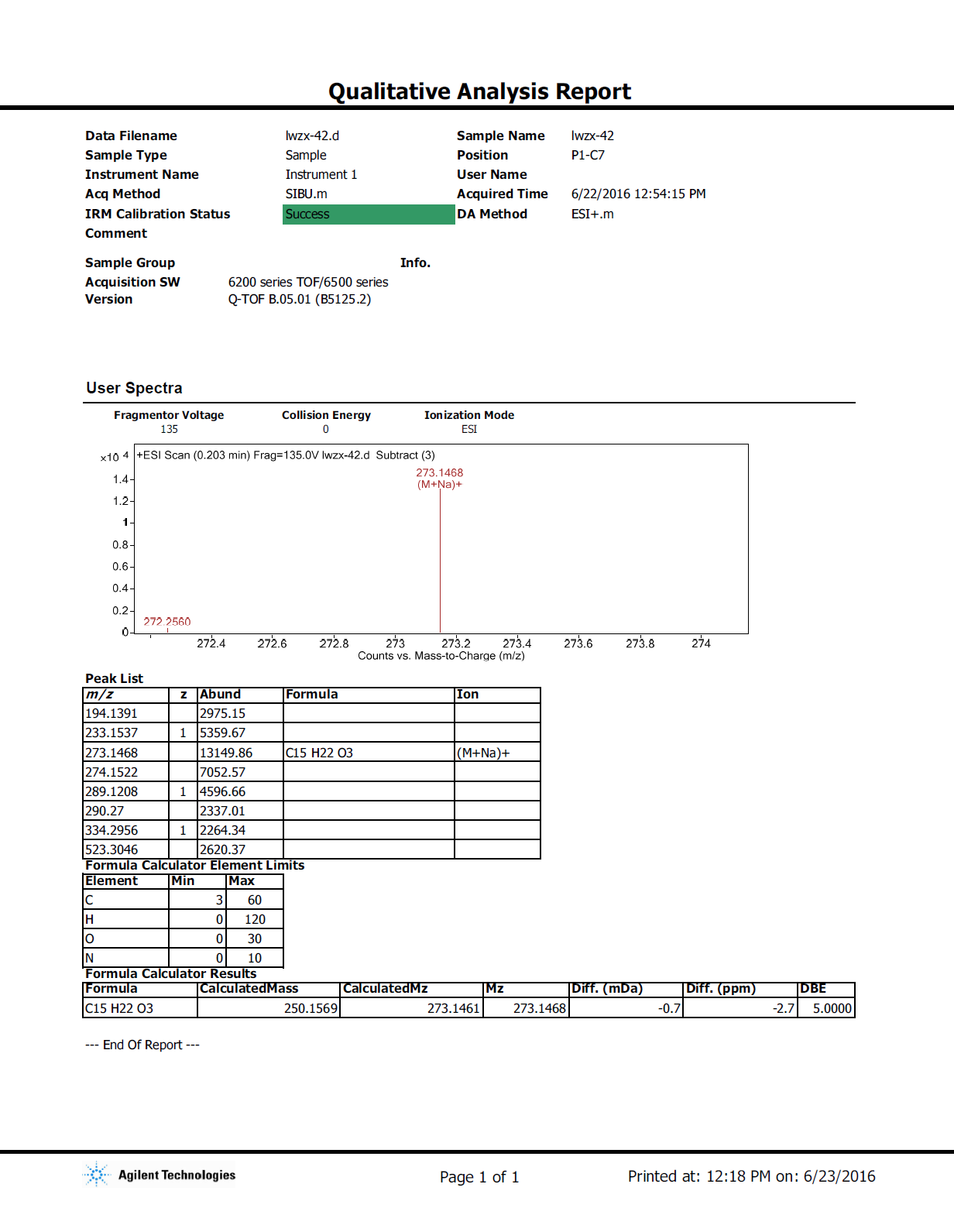


# Figure 48S. ^1^H NMR spectrum of **9** (600 MHz, CDCl_3_).

# Figure 49S. ^13^C NMR and DEPT spectra of **9** (150 MHz, CDCl_3_).

# Figure 50S. HSQC spectrum of **9**.

# Figure 51S. ^1^H-^1^H COSY spectrum of **9**.

# Figure 52S. HMBC spectrum of **9**.

# Figure 53S. ROESY spectrum of **9**.

# Figure 54S. (+)-HRESIMS report of **9**.


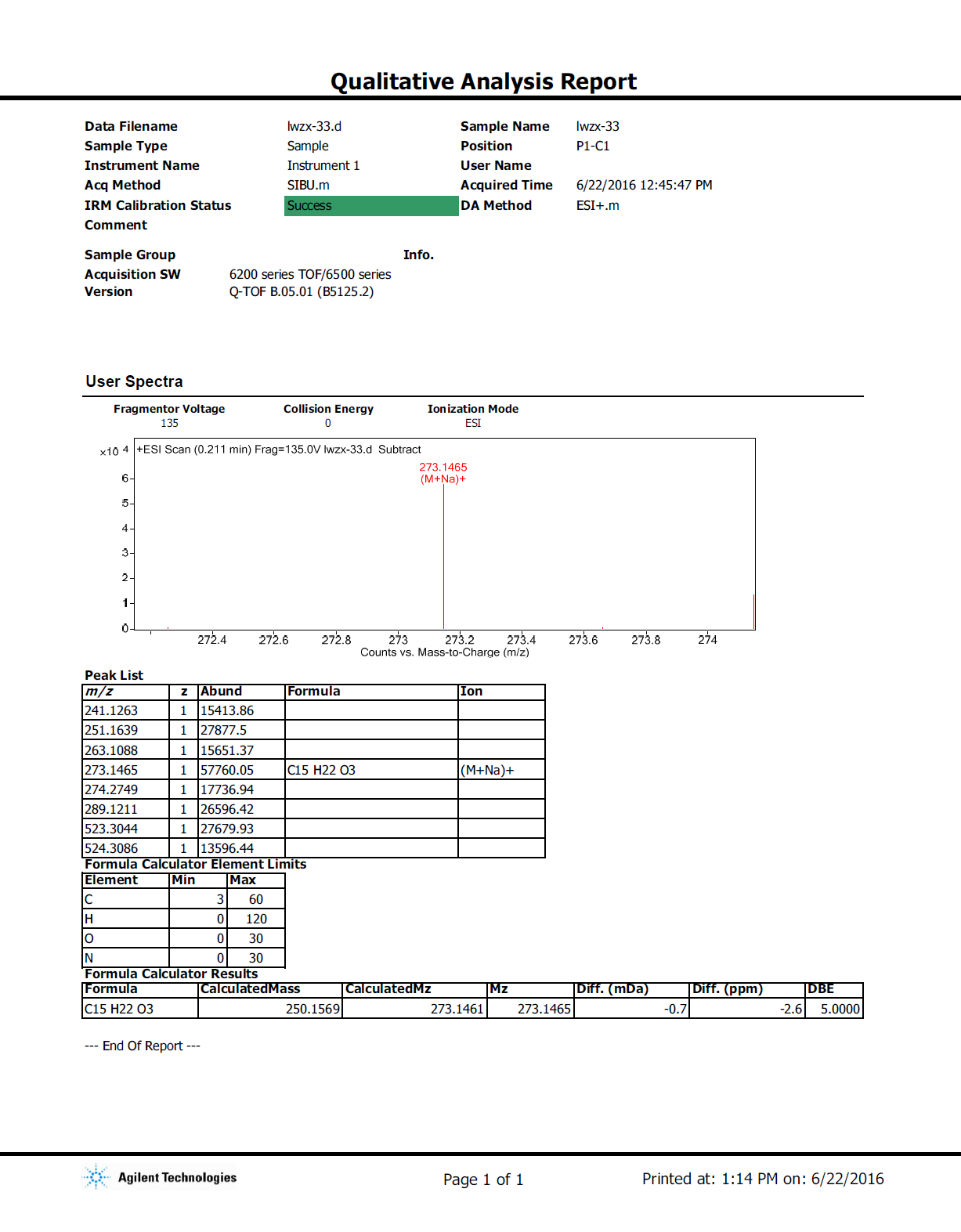


# Figure 55S. ^1^H NMR spectrum of **10** (600 MHz, CDCl_3_).

# Figure 56S. ^13^C NMR spectrum of **10** (150 MHz, CDCl_3_).

# Figure 57S. HMBC spectrum of **10**.

# Figure 58S. ROESY spectrum of **10**.

# Figure 59S. (+)-HRESIMS report of **10**.


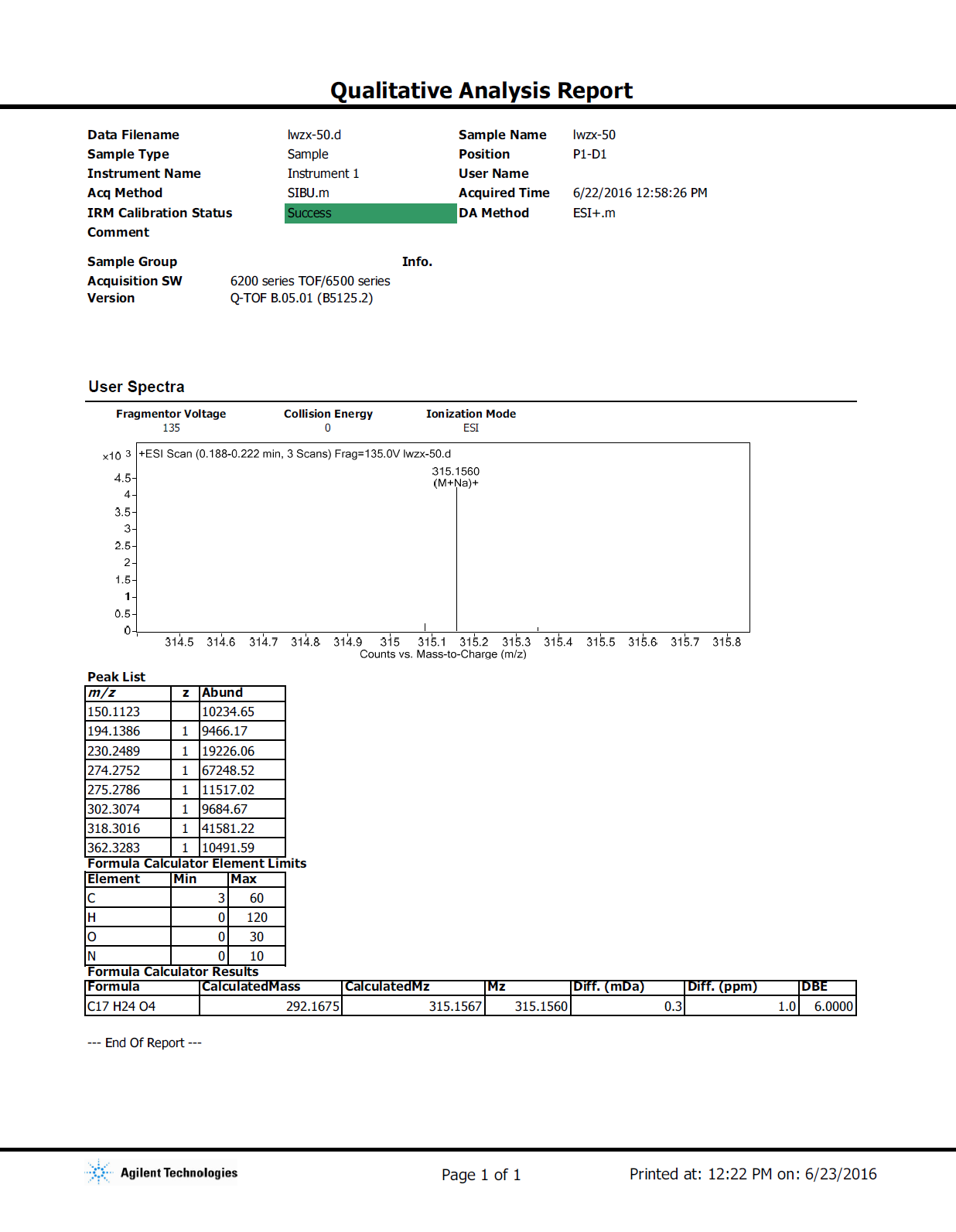


# Figure 60S. ^1^H NMR spectrum of **11** (600 MHz, CDCl_3_).

# Figure 61S. ^13^C NMR and DEPT spectra of **11** (150 MHz, CDCl_3_).

# Figure 62S. HSQC spectrum of **11**.

# Figure 63S. ^1^H-^1^H COSY spectrum of **11**.

# Figure 64S. HMBC spectrum of **11**.

# Figure 65S. ROESY spectrum of **11**.

# Figure 66S. (+)-HRESIMS report of **11**.


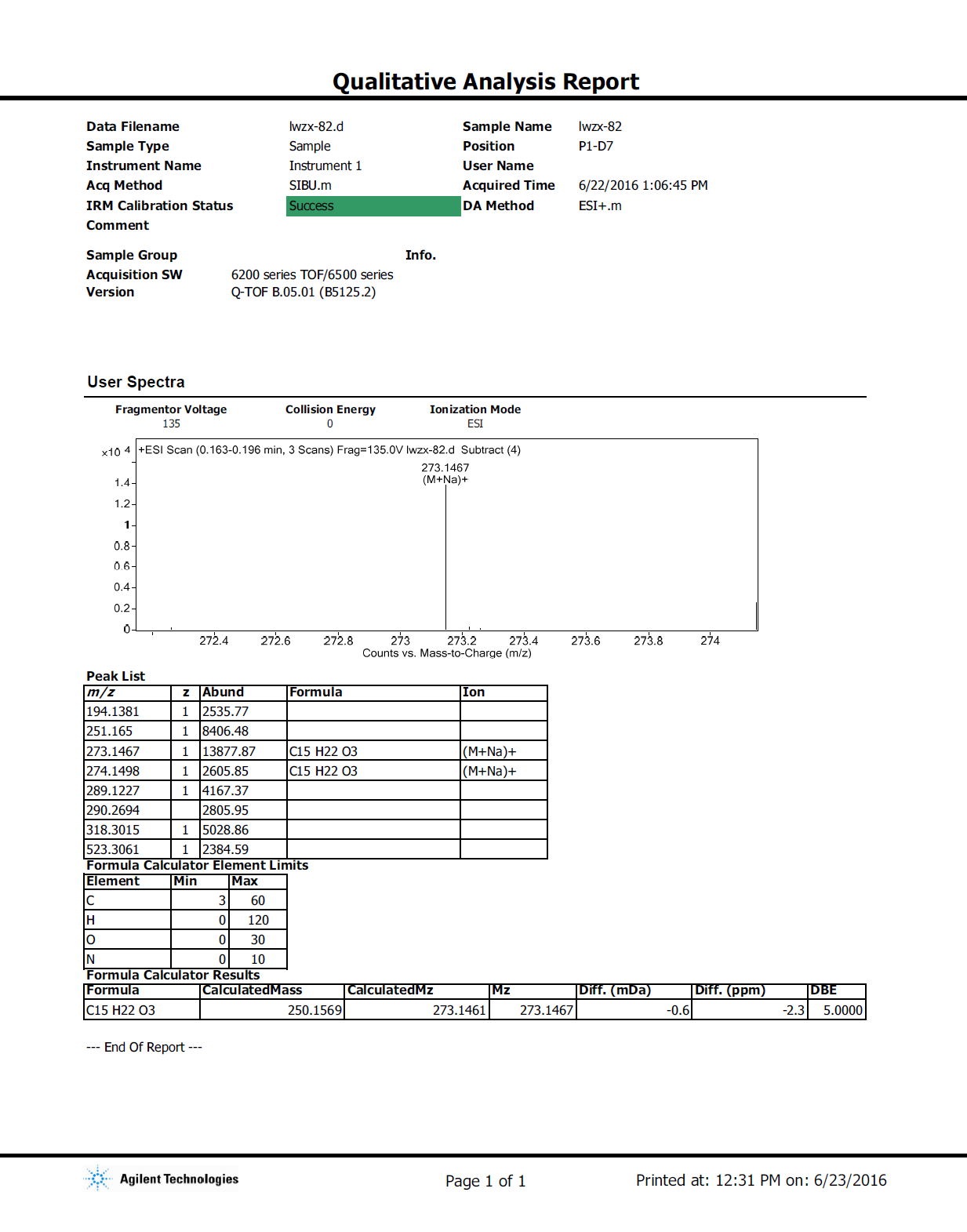


# Figure 67S. ^1^H NMR spectrum of **12** (600 MHz, CDCl_3_).

# Figure 68S. ^13^C NMR and DEPT spectra of **12** (150 MHz, CDCl_3_).

# Figure 69S. HSQC spectrum of **12**.

# Figure 70S. ^1^H-^1^H COSY spectrum of **12**.

# Figure 71S. HMBC spectrum of **12**.

# Figure 72S. ROESY spectrum of **12**.

# Figure 73S. (+)-HRESIMS report of **12**.


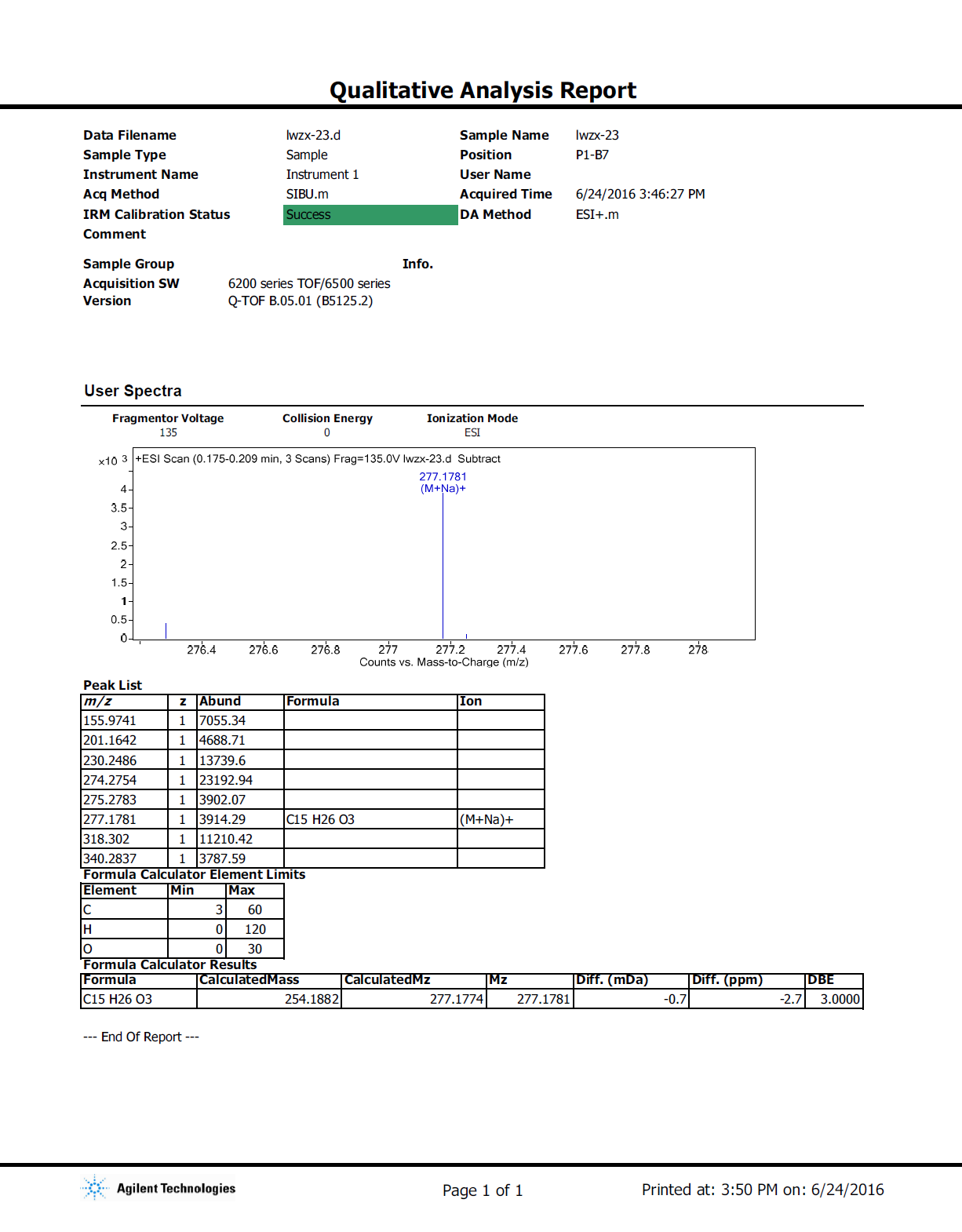


# Figure 74S. ^1^H NMR spectrum of **13** (600 MHz, CDCl_3_).

# Figure 75S. ^13^C NMR and DEPT spectra of **13** (150 MHz, CDCl_3_).

# Figure 76S. HSQC spectrum of **13**.

# Figure 77S. ^1^H-^1^H COSY spectrum of **13**.

# Figure 78S. HMBC spectrum of **13**.

# Figure 79S. ROESY spectrum of **13**.


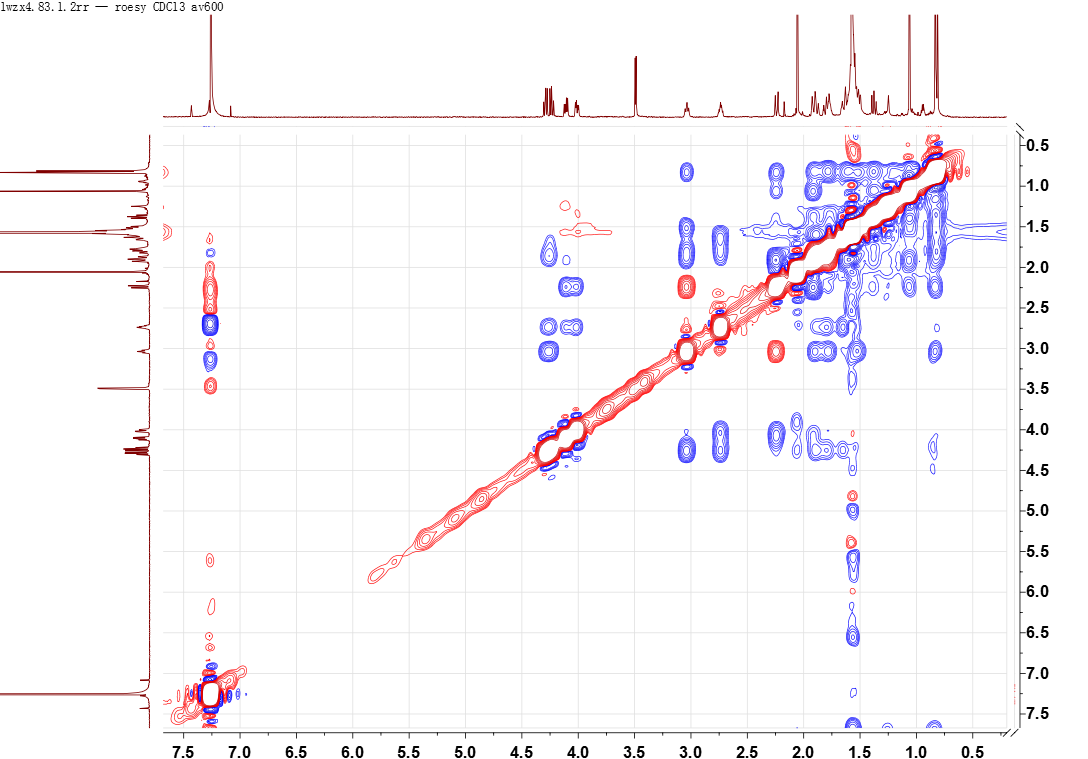


# Figure 80S. (+)-HRESIMS report of **13**.


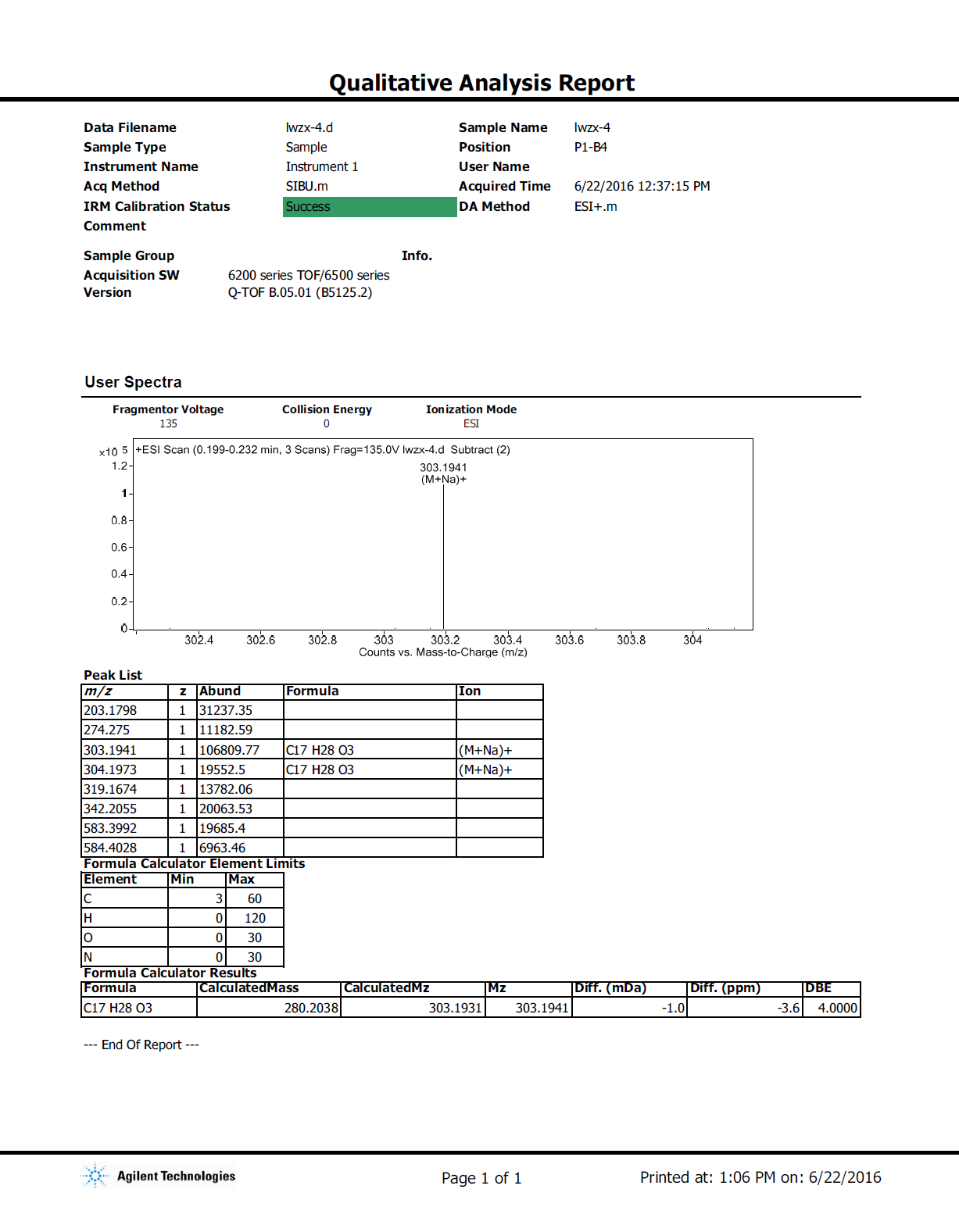


# Figure 84S. ^1^H NMR spectrum of **14** (600 MHz, CDCl_3_).

# Figure 85S. ^13^C NMR and DEPT spectra of **14** (150 MHz, CDCl_3_).

# Figure 86S. HSQC spectrum of **14**.

# Figure 87S. ^1^H-^1^H COSY spectrum of **14**.

# Figure 88S. HMBC spectrum of **14**.

# Figure 89S. ROESY spectrum of **14**.

# Figure 90S. (+)-HRESIMS report of **14**.


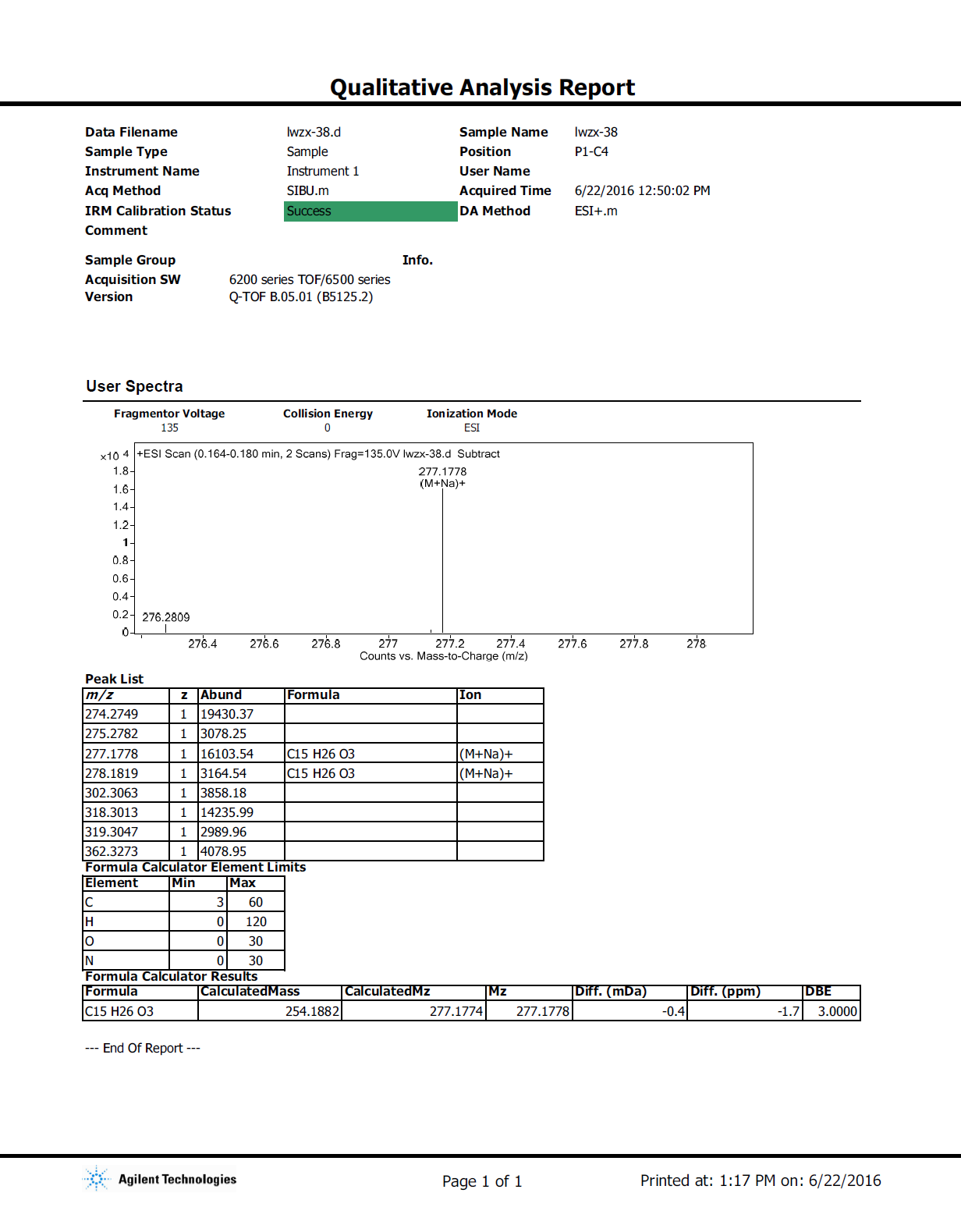


# Figure 81S. ^1^H NMR spectrum of **15** (600 MHz, CDCl_3_).

# Figure 82S. ^13^C NMR spectrum of **15** (150 MHz, CDCl_3_).

# Figure 83S. HSQC spectrum of **15**.

# Figure 84S. ^1^H-^1^H COSY spectrum of **15**.

# Figure 85S. HMBC spectrum of **15**.

# Figure 86S. ROESY spectrum of **15**.

# Figure 87S. (+)-HRESIMS report of **15**.


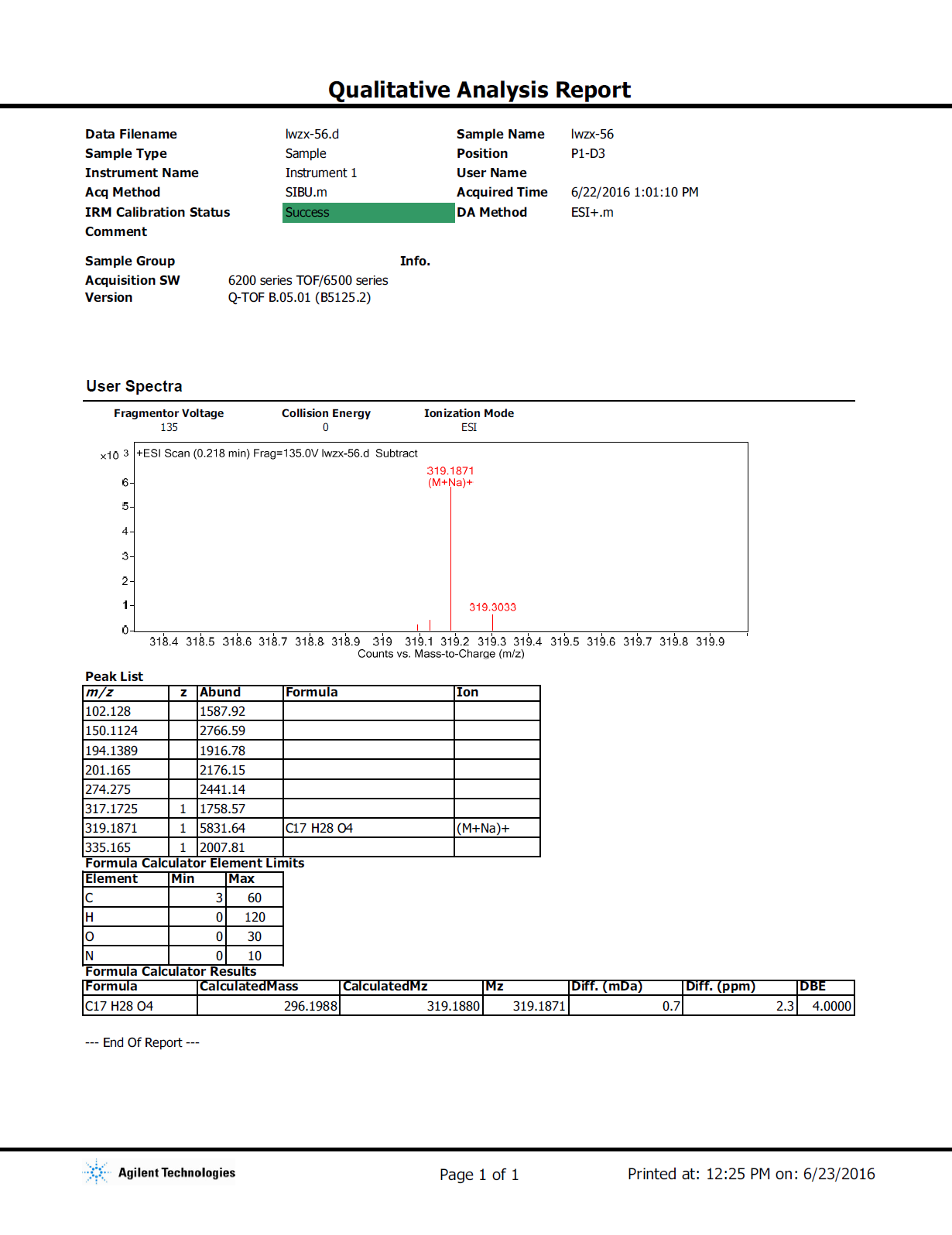

Supplement: Supplementary file 1 — Supplementary file1 (DOCX 26863 kb) [file 13659_2020_239_MOESM1_ESM.docx]
